# The Prevalence, Features, Influencing Factors, and Solutions for COVID-19 Vaccine Misinformation: Systematic Review

**DOI:** 10.2196/40201

**Published:** 2023-01-11

**Authors:** Sihong Zhao, Simeng Hu, Xiaoyu Zhou, Suhang Song, Qian Wang, Hongqiu Zheng, Ying Zhang, Zhiyuan Hou

**Affiliations:** 1 School of Public Health, Global Health Institute National Health Commission Key Laboratory of Health Technology Assessment Fudan University Shanghai China; 2 Department of Health Policy and Management College of Public Health University of Georgia Athens, GA United States

**Keywords:** COVID-19, COVID-19 vaccine, misinformation, anti-vaccine, review, social media, survey

## Abstract

**Background:**

During the COVID-19 pandemic, infodemic spread even more rapidly than the pandemic itself. The COVID-19 vaccine hesitancy has been prevalent worldwide and hindered pandemic exiting strategies. Misinformation around COVID-19 vaccines is a vital contributor to vaccine hesitancy. However, no evidence systematically summarized COVID-19 vaccine misinformation.

**Objective:**

This review aims to synthesize the global evidence on misinformation related to COVID-19 vaccines, including its prevalence, features, influencing factors, impacts, and solutions for combating misinformation.

**Methods:**

We performed a systematic review by searching 5 peer-reviewed databases (PubMed, Embase, Web of Science, Scopus, and EBSCO). We included original articles that investigated misinformation related to COVID-19 vaccines and were published in English from January 1, 2020, to August 18, 2022. We excluded publications that did not cover or focus on COVID-19 vaccine misinformation. The Appraisal tool for Cross-Sectional Studies, version 2 of the Cochrane risk-of-bias tool for randomized trials (RoB 2), and Critical Appraisal Skills Programme Checklist were used to assess the study quality. The review was guided by PRISMA (Preferred Reporting Items for Systematic Reviews and Meta-Analyses) and registered with PROSPERO (CRD42021288929).

**Results:**

Of the 8864 studies identified, 91 observational studies and 11 interventional studies met the inclusion criteria. Misinformation around COVID-19 vaccines covered conspiracy, concerns on vaccine safety and efficacy, no need for vaccines, morality, liberty, and humor. Conspiracy and safety concerns were the most prevalent misinformation. There was a great variation in misinformation prevalence, noted among 2.5%-55.4% in the general population and 6.0%-96.7% in the antivaccine/vaccine hesitant groups from survey-based studies, and in 0.1%-41.3% on general online data and 0.5%-56% on antivaccine/vaccine hesitant data from internet-based studies. Younger age, lower education and economic status, right-wing and conservative ideology, and having psychological problems enhanced beliefs in misinformation. The content, format, and source of misinformation influenced its spread. A 5-step framework was proposed to address vaccine-related misinformation, including identifying misinformation, regulating producers and distributors, cutting production and distribution, supporting target audiences, and disseminating trustworthy information. The debunking messages/videos were found to be effective in several experimental studies.

**Conclusions:**

Our review provides comprehensive and up-to-date evidence on COVID-19 vaccine misinformation and helps responses to vaccine infodemic in future pandemics.

**Trial Registration:**

PROSPERO CRD42021288929; https://tinyurl.com/2prejtfa

## Introduction

### Background

The COVID-19 pandemic has become the most threatening global health issue for almost 3 years [1]. As a cost-effective measure to protect people, governments have implemented various policies to promote the COVID-19 vaccination. However, as of early 2022, the global acceptance rate of the COVID-19 vaccination was only 67.8% [2]. Considering the powerful capability of the omicron variant to escape neutralizing antibodies elicited by current vaccines, the current vaccine acceptance rate is not enough to control the omicron variant [3,4]. It is thus necessary to investigate the negative factors that hinder the COVID-19 vaccination and take actions to increase vaccine coverage.

Vaccine acceptance is determined by contextual influences, individual/social group influences, and vaccine- and vaccination-specific issues [5]. Among all these factors, influences of infodemic and vaccine misinformation deserve more attention for the COVID-19 vaccination. An infodemic is an overabundance of information including misinformation in digital and physical environments, which makes it hard to find trustworthy sources and reliable guidance during a disease outbreak [6]. In the era of social media, the dissemination of information, especially misinformation, has been intensified [7]. During the COVID-19 pandemic, COVID-19 infodemic spread even more rapidly than the pandemic itself. The COVID-19 infodemic had jeopardized public trust in the pandemic response strategies such as vaccination and attracted attention from governments and health agencies across the world [8]. It is difficult but urgent to terminate and resolve the infodemic to promote the vaccination.

Misinformation is referred to false or inaccurate information deliberately intended to deceive [9]. It originates from rumors, websites and social media, works of fiction, governments, politicians, and vested interests [10,11]. Misinformation around COVID-19 vaccines is a noteworthy component of contextual influences on vaccine acceptance or hesitancy [12,13]. It can distort people’s perception of COVID-19 vaccines [14], exaggerate the probability of adverse events following vaccination [15], and lead to extreme political sentiments [16]. COVID-19 vaccine hesitancy, partially driven by misinformation, heavily hindered the pandemic exiting strategies worldwide.

Some reviews have summarized COVID-19–related infodemic and misinformation during the pandemic, but there is a lack of systematic evidence focusing on COVID-19 vaccine misinformation. For instance, Ries [17] synthesized the mechanisms and impacts of COVID-19 infodemic. Gabarron and colleagues [18] summarized the types of COVID-19–related misinformation and its possible consequences. A few studies focused on misinformation about COVID-19 vaccines, but they mainly evaluated its influence on vaccine hesitancy [19,20]. However, no evidence systematically summarized the distribution of COVID-19 vaccine misinformation in the population, what features it has, and how to fight against it.

### Objective

We aimed to synthesize global evidence on misinformation related to COVID-19 vaccines, including its prevalence, features, influencing factors, impacts, and solutions for combating misinformation around COVID-19 vaccines. Specifically, the following questions guided our inquiry: How prevalent was COVID-19 vaccine misinformation across regions and populations? What types and features did the misinformation have? Where did the misinformation come from? How was it distributed among the general population? What factors affected misinformation believing and spreading? How did the misinformation influence vaccine hesitancy and behaviors? How to fight against vaccine misinformation in a future pandemic? This systematic review would enrich the evidence regarding vaccine misinformation and inform response strategies when new vaccines are introduced in future pandemics.

## Methods

### Search Strategy and Selection Criteria

According to the PRISMA (Preferred Reporting Items for Systematic Reviews and Meta-Analyses) guidelines, we conducted a systematic review of empirical articles on COVID-19 vaccine misinformation. The PRISMA checklist can be found in Multimedia Appendix 1. The review protocol was registered with PROSPERO (registration number CRD42021288929). This review was developed based on 5 peer-reviewed databases (PubMed, Embase, Web of Science, Scopus, and EBSCO). We used keywords related to COVID-19 vaccines and misinformation to identify empirical articles published in English from January 1, 2020, to August 18, 2022.

To identify the keywords of “misinformation,” we referred to the codebook by Kata [10], a widely recognized study on the classification of vaccine misinformation. We also referred to the fact sheet of COVID-19 vaccine misinformation published by the US Centers for Disease Control and Prevention (CDC) and collected the keywords on their websites to match the emerging misinformation about COVID-19 vaccines [21]. Using the keywords above, we piloted literature search in PubMed and Web of Science, and further refined the keywords according to literature searching results. The search strategy (Multimedia Appendix 2) consisted of 2 major concepts: COVID-19 vaccine and misinformation, which contains the general descriptions of misinformation (such as “misinformation,” “infodemic,” “myth”) and specified descriptions of some certain misinformation (such as “fertility,” “toxic,” “freedom”).

Original observational or interventional articles that investigated misinformation related to COVID-19 vaccines were included. We excluded studies that (1) investigated non-COVID-19 vaccines or the COVID-19 pandemic instead of COVID-19 vaccines, (2) did not investigate misinformation, and (3) did not focus on COVID-19 vaccine misinformation. We also excluded the following article types: conference abstract, editorial, letter, commentary, correspondence, study protocol, and review.

### Data Screening and Extraction

We exported identified articles from databases, imported them into EndNote 20 (Clarivate Analytics), and removed the duplicates. Two reviewers (SH and SZ) first screened titles and abstracts independently to include articles meeting the inclusion criteria. The full texts of included studies after initial screening were scrutinized to assess the overall eligibility based on the inclusion and exclusion criteria by 2 independent reviewers (SH and SZ). When discrepancies in article inclusion emerged between the 2 reviewers, they engaged in discussion with a third researcher (XZ) to reach a consensus.

For eligible studies, data were independently extracted by 2 reviewers (SH and SZ), and inconsistencies or disagreements were reconciled in data extraction. Besides the study characteristics (region and period, study design, data sources, target population, sample size, and analysis methods), we extracted 4 outcomes of interest for each included study: (1) the types, sources, and prevalence of COVID-19 vaccine misinformation; (2) factors that affect the believing and spreading of COVID-19 vaccine misinformation; (3) the impact of misinformation on vaccine hesitancy and behaviors; and (4) proposed solutions for combating misinformation.

### Classification of Study Types, Populations, and Phases

In this systematic review, we covered 4 types of study design: survey-based study, internet-based study, interview, and experiment. Survey-based study included cross-sectional or follow-up studies conducted among population using questionnaires; and internet-based study referred to studies that acquired publicly available information thought the internet. The prevalence and impact of COVID-19 vaccine misinformation might be different between the vaccine hesitant/refusal group and the general population. Thus, each type of studies was divided into 2 subgroups according to their study population’s prior defined attitudes toward COVID-19 vaccines: the antivaccine/vaccine hesitant group and the general population group.

To investigate the potential change in vaccine misinformation in terms of its types, contents, prevalence, and impact, we used 2 key time points to define 3 phases in this review: prevaccination phase (phase 1), from the outbreak of COVID-19 to the first dose of COVID-19 vaccines being injected at December 8, 2020 [22]; postvaccination and pre-Omicron phase (phase 2), from the end of phase 1 to the date that the new variant Omicron was officially reported (November 26, 2021) [23]; post-Omicron phase (phase 3), after November 26, 2021. If the time frame in data collection covered the time point and the ending/beginning date was not close to the time point (over 1 month), the study phase was considered to have crossed 2 phases.

### Framework of Classifying Misinformation Types and Contents

Like the identification process of misinformation-related keywords, we first adapted the codebook by Kata [10] to construct a framework to classify the types and contents of COVID-19 vaccine misinformation. To cover the emerging and evolving COVID-19 vaccine infodemic, we further referred to the CDC fact sheet to revise the framework [21]. Our primary framework consisted of main classifications and contents from Kata’s work as well as contents from the CDC fact sheet. New types or contents of misinformation may have occurred when we extracted data from the included studies, and would be considered to finalize our framework. The original misinformation contents extracted from articles were rechecked using the final framework. Discrepancies in misinformation classification were discussed and reconciled.

### Framework of Solutions for Combating Misinformation

For clarity and consistency in data extraction, we sorted solutions for combating misinformation into a 5-step framework, which was adapted from the disinfodemic policy brief by the United Nations Educational, Scientific and Cultural Organization (UNESCO) [24]. In this policy brief, solutions to debunk the misinformation consisted of 4-part responses: identifying misinformation, regulating producers and distributors, cutting production and distribution, and supporting the target audiences of misinformation. As the dissemination of trustworthy information deserved more attention, it was added as the fifth part of the framework.

### Quality Assessment

As study designs varied across eligible studies, we used 3 kinds of quality assessment tools to assess their quality. The Appraisal tool for Cross-Sectional Studies (AXIS) was used to evaluate survey-based cross-sectional studies, and its modified version was applied to assess internet-based studies [25]. The version 2 of the Cochrane risk-of-bias tool for randomized trials (RoB 2) tool and the Critical Appraisal Skills Programme Checklist were used to assess methodological quality in experimental studies and interviews, respectively [26,27]. Quality assessment was conducted by 2 reviewers (SH and SZ), and the risk of bias for each eligible study was classified as “low risk,” “some concerns,” or “high risk.” The detailed scoring criteria of quality assessment are shown in Multimedia Appendix 3.

### Patient and Public Involvement

Patients or the public were not involved in the design, or conduct, or reporting, or dissemination plans of our research.

### Ethical Approval

Ethical approval was waived as this is a secondary analysis on the published articles.

## Results

### Basic Characteristics of Included Studies

**Figure 1:**
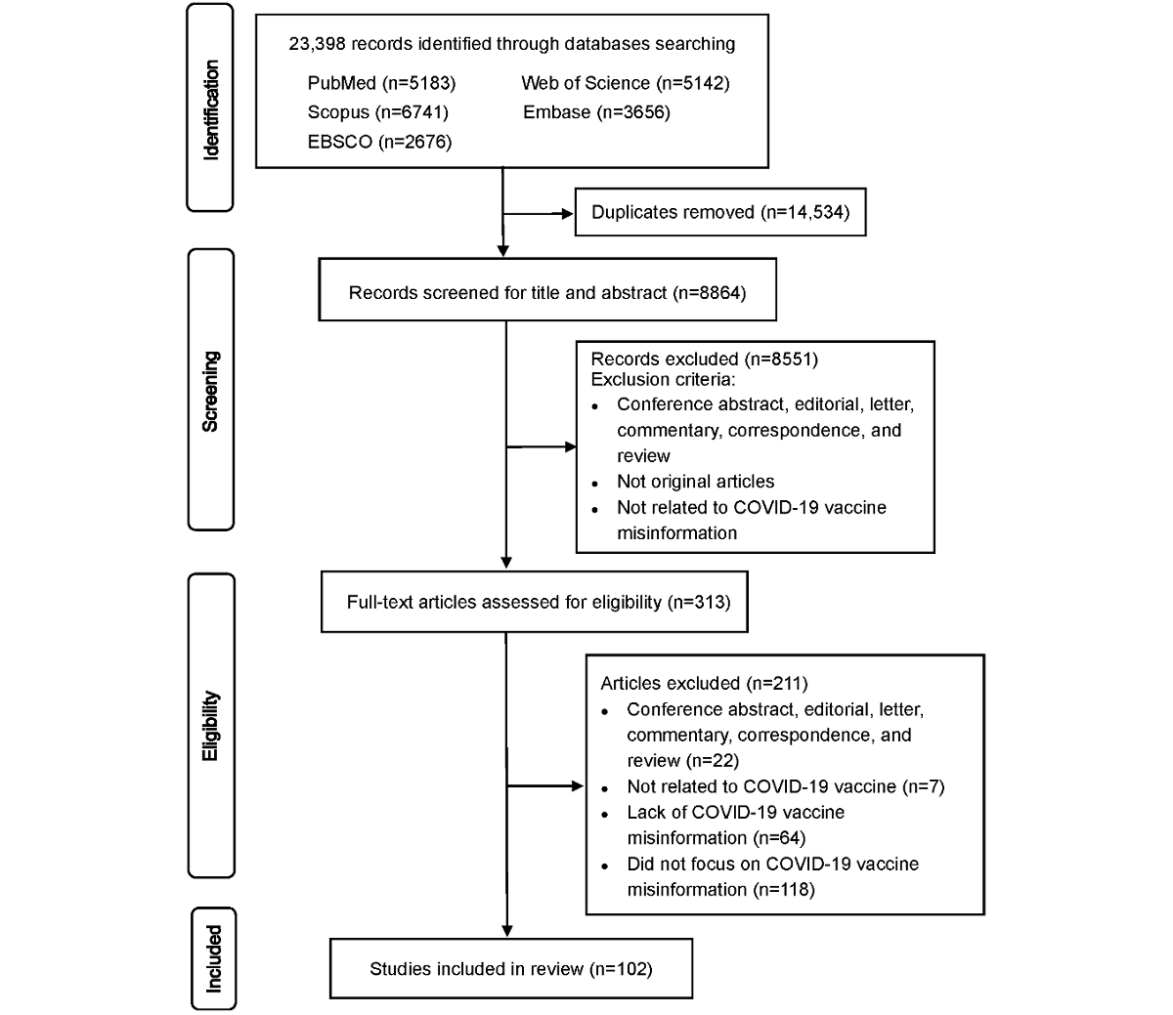
Flow diagram.

In total, we identified 23,398 studies from 5 peer-reviewed databases (Figure 1). After removing duplicates, 8864 studies were initially screened based on their titles and abstracts, and after initial screening, 313 studies underwent full-text assessment for eligibility. A total of 102 studies met the inclusion criteria, including 91 observational studies and 11 interventional studies. According to the quality assessment criteria, the majority (90/102) of included studies were in the low risk of bias category. Eight studies were classified as “some concerns” and 4 were classified as “high risk.” The basic characteristics of included studies are presented in Table 1, and further details about each study are listed in Multimedia Appendix 4 (see also [28-71]).

Among the 91 observational studies, 38 conducted surveys, 42 analyzed data from the internet, 10 used interviews, and the remaining 1 performed mixed methods of cross-sectional survey and interview. In terms of study region, the American region was the most studied (n=25), followed by the European (n=19) and Eastern Mediterranean (n=11) regions. By contrast, the African (n=6), South-East Asian (n=5), and Western Pacific (n=4) regions were the less investigated.

According to the prior defined attitudes toward COVID-19 vaccines in study populations, survey- and internet-based studies can be divided into the following: survey-based study on general population (n=31), survey-based study on antivaccine/vaccine hesitant population (n=4), internet-based study on general online data (n=22), and internet-based study on antivaccine/vaccine hesitant data (n=19). A total of 3 survey-based studies and 1 internet-based study reported results from both general population/data and antivaccine/vaccine hesitant population/data.

**Table 1:** Basic characteristics of included articles.

Study characteristics							Studies, n
**Study type**							
	**Observational study**						
			Survey-based study			38	
			Internet-based study			42	
			Interview			10	
			Mixed methods study			1	
	**Experimental study**						
			Randomized trial			9	
			Quasi trial			2	
**Risk of bias**							
	Low risk					90	
	Some concern					8	
	High risk					4	
**Study phase^a^**							
	No report					8	
	Phase 1					28	
	Phase 2					49	
	Phase 3					1	
**Region**							
	Worldwide^b^					26	
	African					6	
	American					25	
	Eastern Mediterranean					11	
	European					19	
	South-East Asian					5	
	Western Pacific					4	
	More than 1 region^c^					6	
**Prior defined attitudes toward COVID-19 vaccines^d^ (n=80)**							
	**Survey-based study**						
			General population			31	
			Antivaccine/vaccine hesitant population			4	
			Both groups above			3	
	**Internet-based study**						
			General online data			22	
			Antivaccine/vaccine hesitant data			19	
			Both groups above			1	
**Platform for internet-based studies (n=42)**							
	**Social media**						
		Twitter				20	
		YouTube				5	
		Facebook				4	
		TikTok				1	
		Parler				1	
		Multiple social media platforms^e^				3	
Google Trends							1
Google Images							1
Internet news							1
Online article database							1
Multiple platforms^f^							1

^a^Study phase was defined as follows: phase 1, from November 2019 to December 8, 2020; phase 2, from December 8, 2020, to November 26, 2021; phase 3 from November 26, 2021, to the latest ending date of data collection in included studies.

^b^Studies covering worldwide, including internet-based study with no limit in data collection (n=24), and survey-based study in multiple countries (n=2).

^c^Studies covering more than 1 region: 3 studies on American, European, and Western Pacific regions; and another 3 on American and European regions.

^d^Study populations differ by their prior defined attitudes toward COVID-19 vaccines: studies reporting misinformation among the vaccine hesitancy or refusal group (“antivaccine/vaccine hesitant group” and “antivaccine/vaccine hesitant data” in the table), and studies which did not prior define the vaccine hesitancy or refusal group (“general population” and “general online data” in the table).

^e^Multiple social media platforms in 3 studies [28,72,73] were Facebook, Instagram, and Twitter; Instagram and Facebook; and YouTube, Twitter, Facebook, and Instagram.

^f^Multiple platforms included Google, Google Fact Check, Facebook, YouTube, Twitter, fact-checking agency websites, and websites of television and newspaper.

Most internet-based studies used social media platforms as data source, of which the most frequently discussed was Twitter (n=20), followed by YouTube (n=5), Facebook (n=4), TikTok (n=1), and Parler (n=1). Besides social media, 4 studies used general internet information, such as Google and online news database, as data source. A total of 4 studies used data from multiple platforms or multiple social media. Most internet-based studies did not limit the region of data source (n=24). Among those internet-based studies reporting study regions, the Americas (n=8) and Europe (n=6) were more commonly addressed, whereas the Western Pacific (n=2), Eastern Mediterranean (n=1), Africa (n=1) were less studied, leaving no internet-based study in South-East Asia. Most internet-based studies analyzed information written in English (n=33), whereas non-English information were less studied: 3 for Spanish, 2 for Arabic, 1 for both English and Spanish together, and 1 each for Chinese, Italian, and Turkish.

### Types, Sources, and Prevalence of Misinformation

About 90% (91/102, 89.2%) of studies mentioned the types of COVID-19 vaccine–related misinformation. Using the coding framework mentioned in the “Methods” section, we divided vaccine-related misinformation into 7 types: conspiracy, concerns on vaccine safety and efficacy, no need for vaccines, morality, liberty, humor, and overstatement. We further divided these types into 54 different contents (Table 2). Conspiracy, being discussed in 77 studies, was the most commonly studied misinformation, and it could be further specified as vaccine existence conspiracy, political conspiracy, vaccine development and promotion conspiracy, and conspiracy related to a certain group. Concerns on vaccine safety and efficacy, emphasizing the unsubstantiated concerns on safety and efficacy, were the secondary most studied misinformation (n=63). Misinformation about the necessity of vaccines (no need for vaccines) was found in 23 studies, and its subtypes included simple claims such as vaccines are unnecessary, preference of natural immunity and protective behavior, claims about mild COVID-19, COVID-19 denial, underestimation of personal need, and overstatement of vaccine refusal. Misinformation on morality and liberty was found in 15 and 13 studies, respectively; 2 studies investigated humor and another 2 found the overstatement on the effect of COVID-19 vaccines.

Vaccine-related misinformation changed in its contents over time. We compared the misinformation among 3 phases, and found that some conspiracy theories and concerns on vaccine safety circulated in all 3 phases: depopulation, control people, microchip for monitor/control, and financial incentives behind vaccine development in terms of conspiracy; vaccines would alter DNA, and cause fertility, death, or other diseases; the worry of no one would be responsible for potential side effects; beliefs in alternatives such as natural immunity and protection behaviors; referring to vaccines as immoral human experiments and comprising fetal tissue; and individual freedom. Meanwhile, there were emerging and disappearing topics on misinformation. The claims that vaccines were a hoax/fraud only appeared in phase 1. With the progress of COVID-19 vaccination, especially after the massive immunization (phase 2), misinformation about registration (false claims that some vaccines were rejected), against government control (such as fake news that some government will make COVID-19 vaccination mandatory), and conspiracy about vaccine promotion (claims that physicians or people will get financial benefit through promoting vaccination, and that the news about celebrities getting vaccinated are fake: they did not really get vaccinated; rather, they were injected with saline) emerged.

**Table 2:** Types and contents of COVID-19 vaccine–related misinformation.

Type of misinformation and subtype		Explanation	Contents
**Conspiracy**			
	Vaccine existence conspiracy	Conspiracy about the existence of COVID-19 vaccines	No vaccine: COVID-19 vaccines are a conspiracy, hoax, or fraud.
	Political conspiracy	Conspiracy about political purpose related to the vaccines	Device for track/control: Vaccines contain microchip/nanochips and are used for tracking or control people. Population control and new world order: The vaccines are used for the control of population or to create new world order. Depopulation: Vaccines are used for reducing population or for genocide. Bioweapon: Vaccines are a bioweapon. Other conspiracy to a certain subject: Certain people or groups are behind the vaccines for their own good, such as government officials, pharmacy company, Bill Gates.
	Vaccine development and promotion conspiracy	Conspiracy about COVID-19 in vaccine development and vaccination promotion	COVID-19 for vaccines: COVID-19 vaccines existed before the virus, or COVID-19 was made to enforce vaccination. Financial incentive in developing: Vaccines are created only for the profit of pharmaceutical companies and government. Political incentive in developing: Vaccines are approved because of political pressure, or because pharmaceutical company has bought/coerced the government. No enough evidence: Vaccines are untested or not tested enough. Rush in development: Vaccines are rushed in development, thus cannot be trusted. Fabricated vaccine efficacy: Data on vaccine efficacy/effectiveness are fabricated. Cover-up side effect data: Data about the vaccine side effect or death are kept secret. Rejection in registration: Vaccines (which are approved in fact) are rejected in registration for concern. Paid for promotion: Physicians issue vaccines for financial profit. Playacting in promotion: Public figures are vaccinated with inert substances (eg, saline).
	Conspiracy related to a certain group	The conspiracy that vaccines will be harmful, used to control, or be tested in a specific group	Conspiracy of a certain country/region: People in certain country or region are guinea pigs. Vaccines are less effective or is fake in these regions. (eg, African region, Muslim nations). Conspiracy of ethnic minorities: Vaccines are harmful or for killing ethnic minorities; ethnic minorities are guinea pigs (eg, Asian, Black people). Conspiracy of other certain groups: Vaccines are used to reduce these people (eg, elder people, less educated people, low-income group).
**Concerns on vaccine safety and efficacy**			
	Effectiveness	Beliefs that vaccines are ineffective	Vaccines would not work: Vaccines will not work (a simple claim without further explanation). Ineffective in certain groups: Vaccines will not be effective on people with comorbidities such as diabetes, hypertension. Ineffective claim from authority: Government officers or doctors admit vaccines would not work. New strain for cover-up: The new variant of COVID-19 is a hoax and is a cover-up for ineffectiveness of the vaccines. Fake vaccines: Vaccines are water. Same as other vaccines: COVID-9 vaccines are no different from the flu vaccine. Fail before vaccination: Vaccines cannot be preserved properly.
	Safety	Unsubstantiated safety concern	Cause COVID-19: Vaccines will make people catch COVID-19. Worse than COVID-19: Vaccines are more dangerous than the disease itself. Alter DNA: Vaccines will change people’s DNA. Infertility and offspring: Vaccines will make women infertile or affect their offspring. Cause death: Vaccines kill people. Cause other diseases: Vaccines make people get chronic disease, autism, autoimmune disease, paralysis, cancer, physical destruction, impotency, etc. Change people: Vaccines make people magnetic, or turn people into robots, vampires, zombies, etc. Poison: Vaccines contain poisonous materials, such as mercury, toxic ingredients, chemicals. Live virus: COVID-19 vaccines contain a “live strain” of the virus. Unspecified danger: Vaccines are dangerous (a simple claim without further explanation). Side effect responsibility: No one is responsible for the potential side effects of the vaccines.
**No need for vaccines**			
	Unnecessary	A simple claim that COVID-19 vaccines are unnecessary without explanation	Unnecessary: COVID-19 vaccines are not needed.
	Natural immunity and protective behavior	Beliefs that it is better to get natural immunity or get immunity through naturally protective ways, etc.	Alternative: Prefer or believe in natural immunity, herd immunity, or protective behaviors.
	Mild COVID-19	Believe COVID is a mild disease and no need for vaccines	Mild COVID-19: COVID-19 is not dangerous; thus, vaccines are not needed.
	COVID-19 denial	Denial of COVID-19 pandemic leading to COVID-19 vaccines	COVID-19 denial: COVID-19 is a hoax, or a fraud, thus no vaccines are needed.
	Personal need	Beliefs that it is unnecessary for the vaccines because of their health status	Underlying disease: I get autoimmune disease, thus cannot take vaccines. Immune from infection: I am already immune from a past COVID-19 infection. Not a risk group: I am young/healthy/a certain blood type, thus have a low risk of getting COVID-19 or developing serious disease.
	Overstatement of vaccine refusal	Exaggeration about vaccine refusal rate in other places	Refusal exaggeration: 1 in 6 people will refuse COVID-19 vaccines.
**Morality**			
	Religion	Vaccines go against religious belief, or are viewed as a sign of demon	Religion: Vaccines may go against religious beliefs. Devil led people to receive vaccines.
	Human experiment	Vaccine campaign is a human experiment	Human experiment: It is a part of a secret research. Vaccinated ones are guinea pigs.
	Fetal tissue	Fetal remains in vaccines	Fetal tissue: COVID-19 vaccines are made from cells of aborted fetuses.
**Liberty**			
	Against mandatory vaccines/control	False claims about mandatory vaccination, and refusal to obey “control”	Mandatory vaccination: They are forcing us. Against government control: Opposition to the government’s control.
	Support for freedom	Claims that vaccine infringes individual freedom	Freedom: Vaccines are an attempt to take away personal freedom.
	Ignore consent	Concerns that children will be forced to get vaccinated without parents’ consent	Ignore consent: Children getting vaccinated without parental consent.
**Humor**			
	N/A^a^	A humorous but exaggerated way to express unsubstantiated vaccine rumor	Humor: Parody/meme of an adverse reaction.
**Overstatement**			
	Overstatement of the protection/progress of vaccines	Exaggeration about the effect and the scientific progress in COVID-19 vaccines	Overstatement: COVID-19 vaccines are ready; able to cure a patient within 3 hours. After getting the vaccines, we will not have the infection anymore. After getting the COVID-19 vaccines, one can stop wearing the mask and taking safety precaution. As vaccines for COVID-19 have been developed, we can make vaccines for the common cold, HIV, and others.

^a^N/A: not applicable.

Eight studies surveyed the sources of misinformation. Three studies reported social media as main source [74-76]. Two studies found that family and friends also played a role [74,77]. One study measured proportions of different sources: social media (57.6%), followed by family or friends (13.1%), and television (5.7%) [74]. On social media, misinformation was generated majorly by antivaccine groups or well-known antivaccine individuals [78-80]. Two studies found that online celebrity tended to contribute more misinformation [80,81]. One Twitter-based study showed that the highly polarized antivaccine information was mainly from political and nonmedical users, while health care workers were less engaged in COVID-19 vaccine conversation on online platforms [78].

A total of 57 studies reported the prevalence of misinformation (Table 3), including 27 survey-based studies and 30 internet-based studies; further details on the prevalence reported in each study are presented in Multimedia Appendix 5 (see also [29,30,34,36,37,41-43,46-50,52-57,60,62,67,68,70,71]). Among 27 surveys, some investigated both general population and antivaccine groups. The 24 surveys on general population reported a prevalence of general misinformation ranging from 2.5% to 55.4%; concerning the prevalence of specific types of misinformation, conspiracy beliefs varied from 2.5% to 48.4%, concerns about vaccine safety and efficacy from 2.78% to 55.4%, “no need for vaccine” from 3.8% to 28.1%, morality from 1.4% to 20.6%, and liberty from 6% to 36.3%. A total of 6 surveys [29-32,76,82] on antivaccine/vaccine hesitant groups reported a higher prevalence of misinformation: conspiracy, 6.0%-22%; concerns on vaccine safety and efficacy, 12.2%-96.7%; and no need for vaccines, 6.3%-70.4%. The 96.7% prevalence rate was simply driven by 1 study [30] that reported the prevalence on concerns and no need for vaccines; after excluding this outlier, the prevalence of misinformation varied between 6.0% and 20.1%.

Among 30 internet-based studies, 16 investigated general online data and reported the prevalence of general misinformation ranging from 0.1% to 41.3%; for the prevalence of specific types of misinformation, conspiracy ranged from 5.3% to 21.7%, concerns on vaccine safety and efficacy from 0.4% to 11.1%, humor at 26%, no need for vaccines at 10.1%, and vaccine morality from 3.9% to 20.6% (Table 3). Regarding social media platforms, YouTube showed the lowest prevalence of misinformation, ranging from 1.7% to 10.7%, and the prevalence on Twitter varied from 0.4% to 56%; the prevalence of conspiracy is 3% on TikTok and 12.44%-40% on Reddit. Humor accounted for up 26% of all COVID-19 vaccine–related videos in TikTok. In addition, antivaccine/hesitant online posts from the remaining 16 internet-based studies reported a 0.5%-56% prevalence of misinformation; specifically, conspiracy ranged from 3.9% to 55.4%, concerns on vaccine safety and efficacy from 1.3% to 44.8%, no need for vaccines from 0.5% to 3.7%, morality from 2% to 10.4%, and liberty from 5% to 46%.

Some studies reported that the prevalence of misinformation fluctuated over time, and both increasing and decreasing trends were noted [83,84]. One study showed that the change in misinformation was in close association with news or events related to vaccine developments [81].

**Table 3:** Prevalence of COVID-19 vaccine–related misinformation.

Study design and subtype of misinformation		Study, n	Prevalence, %
**Surveys on general population**		24	2.5-55.4
	Conspiracy	22	2.5-48.4
	Concerns on vaccine safety and efficacy	12	2.78-55.4
	No need for vaccines	6	3.8-28.1
	Morality	2	1.4-20.6
	Liberty	2	6-36.3
**Surveys on the antivaccine/vaccine hesitant group**		6	6.0-96.7
	Conspiracy	4	6.0-22
	Concerns on vaccine safety and efficacy	4	12.2-96.7
	No need for vaccines	5	6.3-70.4
**Internet-based studies on general online data**		16	0.1-41.3
	Conspiracy	8	5.3-21.7
	Concerns on vaccine safety and efficacy	3	0.4-11.1
	No need for vaccines	1	10.1
	Morality	2	3.9-20.6
	Liberty	1	41.1
	Humor	1	26
**Internet-based studies on antivaccine/vaccine hesitant data**		15	0.5-56
	Conspiracy	10	3.9-55.4
	Concerns on vaccine safety and efficacy	8	1.3-44.8
	No need for vaccines	3	0.5-3.7
	Morality	2	2-10.4
	Liberty	5	5-46

### Factors That Affect Misinformation Believing and Spreading

In total, 37 articles reported factors that affected the believing and spreading of misinformation. Textbox 1 summarizes individual characteristics and information-seeking behaviors that affect (enhance or reduce) beliefs in misinformation (n=25) as well as misinformation features that promote its spread (n=14).

In terms of geographical areas, 2 studies found a relatively higher prevalence of misinformation in the United States [85,86]. Another study found that Wyoming had the highest level of misinformation in the United States [87]. Living in a village instead of a city was also found to relate to misinformation [88]. Concerning demographic factors, a younger age was found to enhance beliefs in misinformation in 5 studies [89]. The role of sex remained controversial: 5 studies found that females were more likely to accept conspiracy theories than males, while 2 studies found that males were more fragile [90-92]. In the United States, 2 studies found ethnic minorities were related to beliefs in misinformation [93,94], whereas 1 study found that Whites were more susceptible to misinformation than racial/ethnic minorities [91]. Christians or those with a higher level of religiosity were more likely to be influenced by misinformation [91].

Social economic status and occupation also affected beliefs in misinformation. Most studies documented that lower education and economic status were linked to accepting misinformation [89,90,92-100], although 1 study in African and Middle East countries indicated that individuals with higher education levels believed rumors such as changes in human genome due to vaccines [101]. Medical workers were less susceptible to misinformation than the general population in the UK and Jordan, and among medical workers, juniors were more susceptible to misinformation [94,102]. In addition, the unemployed were less likely to trust misinformation than the employed in the UK [91].

For political orientation, right-wing and conservative ideology would increase the conspiracy belief [90,97], and in the United States, Republicans were more likely to accept vaccine conspiracy than Independents [82,103,104]. For disease and migration experience, having basic diseases, no experience of COVID-19 infection or vaccination, and migration could enhance beliefs in misinformation [97,101]. For psychological status and beliefs, depression, perceived ethnic discrimination, national narcissism, and general conspiracy-mindedness were more likely to accept misinformation [93,97,105,106].

**Factors that affect misinformation believing and spreading.**

**Individual characteristics that “enhance” beliefs in misinformation**

Geographic areas:

- United States
- Living in a village instead of a city

Age:

- Younger adults

Sex:

- Mixed influence

Ethnicity:

- Mixed influence in the United States

Religion:

- Christians
- Higher level of religiosity

Socioeconomic status:

- Lower education level
- Lower income
- Lower social economic status

Occupation:

- Employed people

Political orientation:

- Republicans
- Conservatives
- Far-right
- Not being affected or vaccinated

Disease experience:

- Having basic diseases

Migration experience:

- Having migration experience

Psychology status and beliefs:

- Depression
- National narcissism
- Perceived ethnic discrimination
- General conspiracy-mindedness

**Information-related behaviors that “enhance” beliefs in misinformation**

Source of information:

- Social media
- Conservative media
- Family and friends

Awareness of information:

- Feeling less informed about science
- Perceive higher incidence of fake news

Online posting:

- Posting more online

**Individual characteristics and information-related behaviors that “reduce” beliefs in misinformation**

Occupation:

- Medical workers

Channels to accessing information:

- Taking lectures about COVID-19 vaccines
- Trust celebrities for information

**Features of misinformation that “promote” its spread**

Type of misinformation:

- Safety concern
- Conspiracy
- Efficacy concern

Content of misinformation:

- Positive valence (positive emotion)
- Concreteness

Format of misinformation:

- Number of hashtags
- Language or format that mimics news/scientific reports

Source of misinformation:

- Antivaccine group
- Social media influencer
- Health care workers
- Unregulated bot

Platform:

- Different platforms present different misinformation
- Newer social media

The following information-seeking behaviors also enhanced beliefs in misinformation: usage of social media and conservative media [1,92,107,108], posting more online [109], feeling less informed about science [97], and trusting friends or family for COVID-19 information [91]. People who take lectures about COVID-19 vaccines or trust celebrities for COVID-19 information tended to refuse misinformation [91].

In addition, some features of misinformation promoted its spread. Safety concerns, conspiracy, and efficacy concerns were reported as the most popular misinformation types [85,110]. Misinformation posts that had a higher level of positive valence and concreteness would spread [111]. The hashtags and the language or format mimicking news/scientific reports helped misinformation spread [72,110,111]. Misinformation from antivaccine groups, social media influencers, health care workers such as Sherri Tenpenny, and unregulated bots can speed its spread [79,81,112-114]. Each social media has its prevailing misinformation topics [115], and a newer social media platform was more likely to bolster vaccine conspiracy [110].

### Impact of Misinformation on Vaccine Hesitancy and Behaviors

A total of 29 studies indicated that misinformation is related to vaccine hesitancy or negative vaccine perception, and 3 experimental studies also supported this finding [75,91,116]. Misinformation would ignite concerns and fears about the safety profile of vaccines and lead to vaccine hesitancy and refusal [94]. Four studies found a negative relationship between misinformation and vaccine uptake rate [84,87,89,99]. One experimental study in the UK and United States verified that the misinformation exposure significantly reduced both intentions of self-vaccination and vaccination to protect others by around 6% [91]. Skepticism also attenuated the effect of public service messages on promoting vaccination willingness [116]. Besides vaccination behaviors, misinformation reduced the uptake of self-protection behaviors such as mask wearing, distancing, and compliance with health guidance [1,90,107].

The impacts of misinformation may change across different study phases. Impacts of misinformation on protective behaviors were mainly studied in phase 1 (n=4). One study in phase 3 showed a negative relationship between some religious beliefs on COVID-19 vaccines and protective behaviors such as masks and distancing remained [99]. After mass vaccination (phase 2), the negative impact of misinformation on vaccine uptake rate was uncovered and lasted up to phase 3. The negative association of misinformation with attitudes and intention of taking vaccines was consistent among all 3 phases.

### Interventions to Address Misinformation

#### Overview

A total of 65 studies proposed solutions to address COVID-19 vaccine misinformation, and 9 studies assessed the effects of various interventions to combat misinformation. Table 4 summarizes the proposed solutions according to our 5-step framework and specific solutions in each study are detailed in Multimedia Appendix 6 (see also [28, 31, 32, 37-41, 43-45, 48, 50, 52, 54, 56-58, 60, 63-66]).

**Table 4:** Proposed solutions to address COVID-19 vaccine-related misinformation.

Framework and meaning		Detailed solutions	No. of articles	Main actors	Targets
**Identifying misinformation**					
	Identifying misinformation through diverse channels	Routine monitoring and fact-checking Investigation	5	Government and health officers	Misinformation
**Regulating producers and distributors**					
	Regulating the source of misinformation	Legislation and national campaign	3	Political power	Sources of misinformation
**Cutting production and distribution**					
	Cutting the circulation of misinformation	Technical response: more effective moderation policies	5	News media, social media	Misinformation
		Economic response: The boycott of harmful content by advertisers	1	News media, social media	Misinformation
		Curatorial response: dissemination of truth to debunk misinformation	4	News media, social media	The public
**Supporting the target audiences of misinformation**					
	Improving target population’s health literacy and helping the public identify misinformation	Tailored intervention to improve health literacy by suggestion, advocacy, and health education	13	Multiple	Different misinformed groups
		Empowerment by public awareness campaigns and credibility warning labels	3	Multiple	The public
**Disseminating trustworthy information**					
	Implementing multitiered strategies to convey the message by multiple media	N/A^a^	23	Multiple: public health agencies, medical professionals, religious leaders, etc.	The public

^a^N/A: not applicable.

#### Identifying Misinformation

This step contained the routine fact-check and monitoring and investigation of misinformation. A total of 7 articles emphasized that government and health officers should develop a public health surveillance system to track the emergence of misinformation and the outlets of antivaccine groups through data mining applications [97].

#### Regulating Producers and Distributors

Producers and distributors of misinformation need to be regulated by political power. A total of 6 articles mentioned that policy and legal actions should be implemented by the government.

#### Cutting Production and Distribution

This step underlined the reaction to communication platforms, including news media and social media. A total of 11 articles in our review specified this part, which consisted of technical, economic, and curatorial responses. Technical response required social media companies to build and execute more effective moderation policies, such as checking information, altering keyword searches, redirecting individuals to correct sources, banning overt conspiracy groups, and flagging or rapidly removing misinformation [104]. The economic response could be either the boycott of harmful content by advertisers or the monetization limit of the channels producing misinformation [24,108]. The curatorial response emphasized that messages should be directly debunking misinformation. The “backfire effect” (ie, factual counterargument entrenches false beliefs) was found when using debunking messages to address misinformation [117,118]. Therefore, pre-debunking message (inoculation message) before public message communication should be piloted [73,119].

#### Supporting the Target Audiences of Misinformation

A total of 20 studies outlined the importance of supporting the target audiences. Tailored interventions targeting different misinformed groups were considered an important approach to counter the misinformation. Empowerment of the public was recommended to improve health literacy and awareness. Public awareness campaigns were also mentioned in 2 studies [74,102].

#### Disseminating Trustworthy Information

This step seems similar to the curatorial response, yet it focused not only on messages that directly debunk misinformation, but also aimed to pass correct and scientific information. Most studies (n=39) mentioned or exemplified how to disseminate evidence-based information, and they recommended to mobilize trusted medical professionals and scientists to engage in social media conversation. Health care workers and public health agencies should engage in social media and learn how to produce short scientific videos [78,102,120]. Further, collaboration with social media influencers allowed a wider reach to the public [121,122]. Both social media and mass media should be utilized to spread information [123], which can express vaccine support, emphasize scientific procedure, appeal to altruism, picture the meaning and importance of vaccination, create a sense of companionship in the battle of infodemic, or encourage participation through peer pressure. Evidence-based messages should be delivered in ways understandable to individuals from a variety of socioeconomic and educational background. Therefore, youth, religious leaders, community stakeholders, faith-based organizations, and schools could be engaged to co-design culturally compelling and context-appropriate messages [85].

Although many studies proposed solutions, only 9 assessed effects of some interventions to combat misinformation. One observational study detected a limited active impact of the policies developed by Twitter [113]. Among 8 experimental studies, 4 assessed the effect of debunking message/video, 3 assessed the effect of inoculation messages, and 1 assessed the effect of warning tag/cover. All 4 experiments found that debunking message/video could reduce the belief in misinformation [118,124-126]. Yet, 1 experiment found that this effect worked well for people without strong beliefs in misinformation; when it comes to people who strongly believe in misinformation, the result became counterproductive because it may evoke small backfiring effects of vaccination intention [118]. One experiment further showed that partisans exposed to ingroup media (media held the same political preference as participants) perceived debunking messages as more credible and held higher engagement [125]. For inoculation messages, 2 experiments found that simple inoculation message/video could protect people from misinformation [119,127], while the remaining 1 found it had no significant effect, but was useful when combined with viewing or writing comments on the inoculation message [128]. In terms of warning tag/cover, interstitial warnings or cover warnings, which require individuals to click through to continue, were found to be more effective to help participants identify misinformation while warning tags showed no effect [104].

## Discussion

### Principal Findings

This review revealed the features, influencing factors, impacts, and solutions for COVID-19 vaccine misinformation and provided evidence to combat vaccine-related misinformation. The included articles were predominantly from American and European regions, and there was less evidence from African, South-East Asian, and Western Pacific regions. Social media was considered as the main source of COVID-19 vaccine misinformation. Conspiracy, concerns on vaccine safety and efficacy, and no need for vaccines were the most prevalent types of misinformation.

Our review documented the high but wide prevalence of COVID-19 vaccine misinformation. The great variation may be due to the ambiguity in misinformation classification and the difference across social media platforms, regions, and study participants. On social media, antivaccine or misinformation tweets accounted for 0.1%-41.3% of all vaccine-related tweets. Its prevalence varied from 2.5% to 55.4% among the general population, which was consistent with a previous review on general COVID-19 misinformation [18]. Another systematic review also illustrated that the prevalence of misinformation on general vaccines ranged from 1% to 65% on social media between 2012 and 2018 [129]. The prevalence of misinformation posed challenges to the COVID-19 vaccination. As the most prevalent type of COVID-19 vaccine misinformation, conspiracies were usually presented as half-truths, which made it hard to recognize them as misinformation. Therefore, dealing with conspiracy or skepticism should be the priority to combat the prevalent vaccine misinformation.

Social media is considered a “double-edged sword” to inform the public [7]. The endorsement of COVID-19 misinformation was strongly associated with the information sources. People who rely on print media and mainstream print were less apt to endorse COVID-19 misinformation, while the use of social media was positively associated with misperceptions regarding COVID-19 facts. In our review, as the main source of COVID-19 vaccine misinformation, social media usage was related to misinformation believing. Because of its wide usage, social media should be used to debunk misinformation and disseminate trustworthy information [121]. Public health authorities and health professionals should change their low engagement status and be more actively engaged in COVID-19 vaccine conversation on online platforms [78,120]. Social media platforms should also make rules and policies to combat misinformation [113].

The global acceptance of COVID-19 vaccine remains at a low level [2]. Misinformation is negatively associated with vaccine acceptance and self-protection behaviors. In our review, geographic areas, demographic characteristics, education, occupation, political orientation, disease and migration experience, psychological status and beliefs, and information-seeking behaviors were found to influence the believing and spreading of vaccine misinformation. However, there is less clarity on the induced pathway between these factors and misinformation. For example, the higher prevalence of COVID-19 vaccine misinformation in African and West Mediterranean regions was associated with feeling less informed, and the less informed were further linked to poor socioeconomic status, low education level, and lack of information [130]. Future research should thus focus on how these factors influence the believing and spreading of misinformation, and verify targeted populations and intervention strategies to combat vaccine misinformation.

It is urgent to implement effective intervention strategies to combat COVID-19 vaccine misinformation. Through the solutions proposed by 65 included studies, we constructed a 5-step framework to address vaccine-related misinformation. The dissemination of trustworthy information was the most frequently mentioned, followed by supporting target audiences of misinformation and cutting its production and distribution, whereas identifying misinformation and regulating its producers and distributors were less mentioned. However, most proposed solutions were not verified regarding their effects on misinformation. With the limited experimental studies, the debunking message/video was considered as effective interventions against misinformation during the COVID-19 pandemic [118,124-126].

Combating misinformation is a persistent and complex work. More scientific evidence is needed to support misinformation surveillance, punishment of misinformation producers, and dissemination of trustworthy information [24]. As many countries reported the first case of mpox, another epidemic in the shadow of the COVID-19 pandemic is looming [131]. In this context, it is particularly important to synthesize potential intervention strategies to combat vaccine misinformation for future anti-infodemic campaign.

### Limitations

Our review has several limitations. First, most included studies were observational, leading to low quality of evidence. More interventional studies are needed to evaluate the effect of misinformation interventions and identify effective interventions. Second, we only included publications in English, and social media platforms in non-English languages were not included in our review. The feature, prevalence, and impact of COVID-19 vaccine misinformation may differ by languages. Third, nearly one-half of the included articles were based on the internet; however, the internet penetration rates are low in low-income countries. Our review may thus not provide enough data and evidence of COVID-19 vaccine misinformation in low-income countries. Fourth, the included studies were published from January 2020 to August 2022, therefore this period may be insufficient to observe and explore the long-term impact of COVID-19 vaccine misinformation.

### Conclusion

Our review provides comprehensive and up-to-date evidence on COVID-19 vaccine misinformation and helps responses to vaccine infodemic in future pandemics. Its prevalence was high but widely varied worldwide. The most frequent misinformation types were conspiracy and concerns about vaccine safety and efficacy. Information features, information-seeking behaviors, and demographic factors influenced the spreading and believing of misinformation. More evidence is needed to verify potential intervention strategies to combat vaccine misinformation.

## Appendix

Multimedia Appendix 1 PRISMA (Preferred Reporting Items for Systematic Reviews and Meta-Analyses) checklist.

Multimedia Appendix 2 Search strategy for 5 peer-reviewed databases.

Multimedia Appendix 3 Quality assessment criteria.

Multimedia Appendix 4 Characteristics of included studies.

Multimedia Appendix 5 Prevalence of COVID-19 vaccine–related misinformation by study design.

Multimedia Appendix 6 Proposed solutions to address misinformation in each study.

## Abbreviations

AXIS: Appraisal tool for Cross-Sectional Studies
CDC: Centers for Disease Control and Prevention
PRISMA: Preferred Reporting Items for Systematic Reviews and Meta-Analyses
RoB 2: version 2 of the Cochrane risk-of-bias tool for randomized trials
UNESCO: United Nations Educational, Scientific and Cultural Organization
