# Supplementary material for: The Prevalence, Features, Influencing Factors, and Solutions for COVID-19 Vaccine Misinformation: Systematic Review
Source: JMIR Public Health Surveill. 2023 Jan 11;9:e40201. doi: 10.2196/40201 (PMC9838721; doi:10.2196/40201)
Supplement: Multimedia Appendix 2 [file publichealth_v9i1e40201_app2.docx]

## Appendix2. Search strategy for five peer-reviewed databases

| Database | Step | Searching strategy | Number of articles | |
| --- | --- | --- | --- | --- |
| PubMed | #1 | “COVID vaccin*”[Title/Abstract] OR “COVID-19 vaccin*”[Title/Abstract] OR “coronavirus vaccin*”[Title/Abstract] OR “coronavirus disease vaccin*”[Title/Abstract] OR “nCoV vaccin*”[Title/Abstract] OR “2019-nCoV vaccin*”[Title/Abstract] OR “SARS-CoV-2 vaccin*”[Title/Abstract] OR “severe acute respiratory syndrome coronavirus 2 vaccin*”[Title/Abstract] OR “vaccin* for COVID*”[Title/Abstract] OR “vaccin* for coronavirus”[Title/Abstract] OR “vaccin* against COVID*”[Title/Abstract] OR “vaccin* against coronavirus”[Title/Abstract] | 17,772 | |
|  | #2 | “COVID-19 Vaccines” [MeSH Terms] | 14,659 | |
|  | #3 | "information"[Title/Abstract] OR "communication"[Title/Abstract] OR "propaganda"[Title/Abstract] OR "infodemi*"[Title/Abstract] OR "infoveillance"[Title/Abstract] OR "misinformation"[Title/Abstract] OR "disinformation"[Title/Abstract] OR "rumor*"[Title/Abstract] OR "sarcasm*"[Title/Abstract] OR "joke*"[Title/Abstract] OR "humor*"[Title/Abstract] OR "false*"[Title/Abstract] OR "fake*"[Title/Abstract] OR "misleading"[Title/Abstract] OR "unverified"[Title/Abstract] OR "unscientific"[Title/Abstract] OR "myth*"[Title/Abstract] OR "conspirac*"[Title/Abstract] OR "search for truth"[Title/Abstract] OR "suspicion*"[Title/Abstract] OR "skeptic*"[Title/Abstract] OR "vaccine fraud*"[Title/Abstract] OR "vaccine hoax*"[Title/Abstract] OR "vaccine lie*"[Title/Abstract] OR "vaccine scam*"[Title/Abstract] OR "anti-vaccin*"[Title/Abstract] OR "anti-vax*"[Title/Abstract] OR "anti-scien*"[Title/Abstract] OR "liberty"[Title/Abstract] OR "freedom"[Title/Abstract] OR "independence"[Title/Abstract] OR "individuality"[Title/Abstract] OR "human right*"[Title/Abstract] OR "religio*"[Title/Abstract] OR "toxic"[Title/Abstract] OR "poison*"[Title/Abstract] OR "vaccine shedding"[Title/Abstract] OR "abort*"[Title/Abstract] OR "fertility"[Title/Abstract] OR "biological weapon*"[Title/Abstract] OR "autism"[Title/Abstract] | 2,661,726 | |
|  | #4 | "communication"[MeSH Terms] OR "propaganda"[MeSH Terms] OR "information dissemination"[MeSH Terms] OR "information management"[MeSH Terms] OR "health information management"[MeSH Terms] OR "health information exchange"[MeSH Terms] OR "anti-vaccination movement"[MeSH Terms] OR "freedom"[MeSH Terms] OR "human rights"[MeSH Terms] OR "personal autonomy"[MeSH Terms] OR "religion"[MeSH Terms] | 743,166 | |
|  | #5 | English[Language] | 29,699,138 | |
|  | #6 | ("2020/01/01"[Date - Publication]: "2022/08/18"[Date - Publication]) | 4,116,237 | |
|  | #7 | **(#1 OR #2) AND (#3 OR #4) AND #5 AND #6** | **5,183** | |
| Web of Science | #1 | TS=(“COVID vaccin*” OR “COVID-19 vaccin*” OR “coronavirus vaccin*” OR “coronavirus disease vaccin*” OR “nCoV vaccin*” OR “2019-nCoV vaccin*” OR “SARS-CoV-2 vaccin*” OR “severe acute respiratory syndrome coronavirus 2 vaccin*” OR “vaccin* for COVID*” OR “vaccin* for coronavirus” OR “vaccin* against COVID*” OR “vaccin* against coronavirus”) | 26,878 | |
|  | #2 | TS=("information" OR "communication" OR "propaganda" OR "infodemi*" OR "infoveillance" OR "misinformation" OR "disinformation" OR "rumor*" OR "sarcasm*" OR "joke*" OR "humor*" OR "false*" OR "fake*" OR "misleading" OR "unverified" OR "unscientific" OR "myth*" OR "conspirac*" OR "search for truth" OR "suspicion*" OR "skeptic*" OR "vaccine fraud*" OR "vaccine hoax*" OR "vaccine lie*" OR "vaccine scam*" OR "anti-vaccin*" OR "anti-vax*" OR "anti-scien*" OR "liberty" OR "freedom" OR "independence" OR "individuality" OR "human right*" OR "religio*" OR "toxic" OR "poison*" OR "vaccine shedding" OR "abort*" OR "fertility" OR "biological weapon*" OR "autism") | 15,944,021 | |
|  | #3 | **#1AND #2 AND #3**  2022 or 2020 (Publication Years) and Web of Science Core Collection (Database) and English (Languages) | **5,142** | |
| Scopus | #1 | TITLE-ABS-KEY ("COVID vaccin*" OR "COVID-19 vaccin*" OR "coronavirus vaccin*" OR "coronavirus disease vaccin*" OR "nCoV vaccin*" OR "2019-nCoV vaccin*" OR "SARS-CoV-2 vaccin*" OR "severe acute respiratory syndrome coronavirus 2 vaccin*" OR "vaccin* for COVID*" OR "vaccin* for coronavirus" OR "vaccin* against COVID*" OR "vaccin* against coronavirus") | 26,528 |  |
|  | #2 | TITLE-ABS-KEY ( information OR communication OR propaganda OR infodemi* OR infoveillance OR misinformation OR disinformation OR rumor* OR sarcasm* OR joke* OR humor* OR false* OR fake* OR misleading OR unverified OR unscientific OR myth* OR conspirac* OR "search for truth" OR suspicion* OR skeptic* OR "vaccine fraud*" OR "vaccine hoax*" OR "vaccine lie*" OR "vaccine scam*" OR anti-vaccin* OR anti-vax* OR anti-scien* OR liberty OR freedom OR independence OR individuality OR "human right*" OR religio* OR toxic OR poison* OR "vaccine shedding" OR abort* OR fertility OR "biological weapon*" OR autism ) | 10,130,351 |  |
|  | #3 | **#1 AND #2 AND #3**  (LIMIT-TO (PUBYEAR, 2022) OR LIMIT-TO (PUBYEAR, 2020)) AND (LIMIT-TO (LANGUAGE, "English")) | **6,741** |  |
| Embase | #1 | information OR communication OR propaganda OR infodemi* OR infoveillance OR misinformation OR disinformation OR rumor* OR sarcasm* OR joke* OR humor* OR false* OR fake* OR misleading OR unverified OR unscientific OR myth* OR conspirac* OR 'search for truth' OR suspicion* OR skeptic* OR 'vaccine fraud*' OR 'vaccine hoax*' OR 'vaccine lie*' OR 'vaccine scam*' OR anti-vaccin* OR anti-vax* OR anti-scien* OR liberty OR freedom OR independence OR individuality OR 'human right*' OR religio* OR toxic OR poison* OR 'vaccine shedding' OR abort* OR fertility OR 'biological weapon*' OR autism | 3,407,062 |  |
|  | #2 | Information OR 'interpersonal communication' OR misinformation OR humour OR freedom OR 'human rights' OR independence OR individuality OR religion | 2,013,459 |  |
|  | #3 | 'COVID vaccin*' or 'COVID-19 vaccin*' or 'coronavirus vaccin*' or 'coronavirus disease vaccin*' or 'nCoV vaccin*' or '2019-nCoV vaccin*' or 'SARS-CoV-2 vaccin*' or 'severe acute respiratory syndrome coronavirus 2 vaccin*' or 'vaccin* for COVID*' or 'vaccin* for coronavirus' or 'vaccin* against COVID*' or 'vaccin* against coronavirus' | 20,796 |  |
|  | #4 | 'SARS-CoV-2 vaccine' | 1,872 |  |
|  | #5 | [english]/lim AND [embase]/lim AND [2020-2022]/py | 3,496,381 |  |
|  | #6 | **(#1 OR #2) AND (#3 OR #4)** | **3,656** |  |
| EBSCO | #1 | "information" OR "communication" OR "propaganda" OR "infodemi*" OR "infoveillance" OR "misinformation" OR "disinformation" OR "rumor*" OR "sarcasm*" OR "joke*" OR "humor*" OR "false*" OR "fake*" OR "misleading" OR "unverified" OR "unscientific" OR "myth*" OR "conspirac*" OR "search for truth" OR "suspicion*" OR "skeptic*" OR "vaccine fraud*" OR "vaccine hoax*" OR "vaccine lie*" OR "vaccine scam*" OR "anti-vaccin*" OR "anti-vax*" OR "anti-scien*" OR "liberty" OR "freedom" OR "independence" OR "individuality" OR "human right*" OR "religio*" OR "toxic" OR "poison*" OR "vaccine shedding" OR "abort*" OR "fertility" OR "biological weapon*" OR "autism" | 4,053,082 |  |
|  | #2 | “COVID vaccin*” OR “COVID-19 vaccin*” OR “coronavirus vaccin*” OR “coronavirus disease vaccin*” OR “nCoV vaccin*” OR “2019-nCoV vaccin*” OR “SARS-CoV-2 vaccin*” OR “severe acute respiratory syndrome coronavirus 2 vaccin*” OR “vaccin* for COVID*” OR “vaccin* for coronavirus” OR “vaccin* against COVID*” OR “vaccin* against coronavirus” | 43,067 |  |
|  | #3 | **#1 AND #2** | **2,676** |  |
|  |  | Limiters - Published Date: 20200101-20220818 |  |  |
|  |  | Expanders - Apply equivalent subjects |  |  |
|  |  | Narrow by Language: - english |  |  |
|  |  | Search modes - Boolean/Phrase |  |  |
|  |  | Database: Academic Search Complete, Business Source Complete, APA PsycArticles , CINAHL Complete, EconLit, Atla Religion Database with AtlaSerials, MEDLINE, OpenDissertations |  |  |
