# Supplementary material for: The Prevalence, Features, Influencing Factors, and Solutions for COVID-19 Vaccine Misinformation: Systematic Review
Source: JMIR Public Health Surveill. 2023 Jan 11;9:e40201. doi: 10.2196/40201 (PMC9838721; doi:10.2196/40201)
Supplement: Multimedia Appendix 3 [file publichealth_v9i1e40201_app3.docx]

## Appendix3. Quality assessment criteria

## Table S1. Scoring system for surveys using AXIS tool

| Appraisal items | | Maximum score | Individual score | |
| --- | --- | --- | --- | --- |
|  |  |  | **0** | **1** |
| Introduction | | | | |
| 1 | The objective(s) of the study was clear | 1 | No/Don’t know | Yes |
| Methods | | | | |
| 2 | The study design for the stated objective(s) as appropriate | 1 | No/Don’t know | Yes |
| 3 | The sample size was justified | 1 | No: the method of estimating the sample size was not mentioned, or it was mentioned but the limitations of discussion referred to the sample size was not large enough | Yes: the method of estimating the sample size was mentioned |
| 4 | The target population was clearly defined | 1 | No/Don’t know | Yes |
| 5 | The sample frame was taken from an appropriate population base | 1 | No/Don’t know | Yes: the source population-based on sampling was consistent with the target population |
| 6 | The selection process was likely to select participants that were representative of the target population | 1 | No/Don’t know | Yes: the sampling method and sampling process were adequate without obvious bias |
| 7 | Measures were undertaken to address and categorize non-responders | 1 | No/Don’t know | Yes |
| 8 | The risk factor and outcome variables were measured appropriately | 1 | No/Don’t know | Yes |
| 9 | The risk factor and outcome variables were measured correctly using instruments that had been trialed, piloted, or published previously | 1 | No/Don’t know | Yes |
| 10 | The determinant of statistical significance and/or precision estimates is clear | 1 | No/Don’t know | Yes |
| 11 | The methods were sufficiently described to enable them to be repeated | 1 | No/Don’t know | Yes |
| Results | | | | |
| 12 | The basic data was adequately described | 1 | No/Don’t know | Yes |
| 13 | The response rate raise does not concern non-response bias | 1 | No: the response rate was less than 85%, or did not report it | Yes: the response rate was more than 85% |
| 14 | The information about non-responders described | 1 | No/Don’t know | Yes, or did not need to non-response |
| 15 | The results were internally consistent | 1 | No/Don’t know | Yes |
| 16 | The results of the analyses described in the methods were presented | 1 | No/Don’t know | Yes |
| Discussion | | | | |
| 17 | The authors’ discussions and conclusions were justified by the results | 1 | No/don’t know | Yes |
| 18 | The limitations of the study were discussed | 1 | No/Don’t know | Yes |
| Others | | | | |
| 19 | No existence of funding sources or conflicts of interest | 1 | No/Don’t know | Yes |
| 20 | Ethical approval or consent of participants was attained | 1 | No/Don’t know | Yes |
|  | Total | 20 | | |

20~13: low risk; 12~9: some concern; 8 and below: “high risk”.

## Table S2. Scoring system for social-media-based study using AXIS tool

| Appraisal items | | Maximum score | Individual score | |
| --- | --- | --- | --- | --- |
|  |  |  | **0** | **1** |
| Introduction | | | | |
| 1 | The objective(s) of the study was clear | 1 | No/Don’t know | Yes |
| Methods | | | | |
| 2 | The study design for the stated objective(s) as appropriate | 1 | No/Don’t know | Yes |
| 3 | The sample size was justified | NA | - | - |
| 4 | The target population was clearly defined | 1 | No/Don’t know | Yes |
| 5 | The sample frame was taken from an appropriate population base | 1 | No/Don’t know | Yes |
| 6 | The selection process was likely to select participants that were representative of the target population | NA | - | - |
| 7 | Measures were undertaken to address and categorize non-responders | NA | - | - |
| 8 | The risk factor and outcome variables were measured appropriately | 1 | No/Don’t know | Yes |
| 9 | The risk factor and outcome variables were measured correctly using instruments that had been trialed, piloted, or published previously | 1 | No/Don’t know | Yes: used published methods/codebook for coding, used Cohen's kappa coefficient (κ) to measure inter-rater reliability, or did a pilot study |
| 10 | The determinant of statistical significance and/or precision estimates is clear | 1 | No/Don’t know | Yes |
| 11 | The methods were sufficiently described to enable them to be repeated | 1 | No/Don’t know | Yes |
| Results | | | | |
| 12 | The basic data was adequately described | 1 | No/Don’t know | Yes |
| 13 | The response rate raise does not concern non-response bias | NA | - | - |
| 14 | The information about non-responders described | NA | - | - |
| 15 | The results were internally consistent | 1 | No/Don’t know | Yes |
| 16 | The results of the analyses described in the methods were presented | 1 | No/Don’t know | Yes |
| Discussion | | | | |
| 17 | The authors’ discussions and conclusions were justified by the results | 1 | No/Don’t know | Yes |
| 18 | The limitations of the study were discussed | 1 | No/Don’t know | Yes |
| Others | | | | |
| 19 | No existence of funding sources or conflicts of interest | 1 | No/Don’t know | Yes |
| 20 | Ethical approval or consent of participants was attained | NA | - | - |
|  | Total | 15 | | |

15~12: low risk; 11~10: some concern; 8 and below: “high risk”.

## Table S3. Cochrane RoB 2.0 tool used in experimental studies

| Appraisal items | | Maximum score | Individual score | |
| --- | --- | --- | --- | --- |
|  |  |  | 1 | 0 |
| **Bias arising from the randomization process** | | | | |
| 1 | Was the allocation sequence random? | 1 | Yes/ Probably Yes | No/ Probably No/ No information |
| 2 | Was the allocation sequence concealed until participants were enrolled and assigned to interventions? | 1 | Yes/ Probably Yes | No/ Probably No/ No information |
| 3 | Did baseline differences between intervention groups suggest a problem with the randomization process? | 1 | No/ Probably No | Yes/ Probably Yes/ No information |
| **Bias due to deviations from intended interventions** | | | | |
| 4 | Were participants aware of their assigned intervention during the trial? | 1 | No/ Probably No | Yes/ Probably Yes/ No information |
| 5 | Were carers and people delivering the interventions aware of participants’ assigned intervention during the trial? | 1 | No/ Probably No | Yes/ Probably Yes/ No information |
|  | If Yes/ Probably Yes/ No Information to 4 or 5: Were there deviations from the intended intervention that arose because of the trial context? |  | No/ Probably No | Yes/ Probably Yes/ No information |
|  | If Yes/ Probably Yes/ No Information to the question above: Were these deviations likely to have affected the outcome? |  | No/ Probably No | Yes/ Probably Yes/ No information |
|  | If Yes/ Probably Yes to the question above: Were these deviations from the intended intervention balanced between groups? |  | Yes/ Probably Yes | No/ Probably No/ No information |
| 6 | Was an appropriate analysis used to estimate the effect of assignment to intervention? | 1 | Yes/ Probably Yes | No/ Probably No/ No information |
|  | If No/ Probably No/ No Information to 6: Was there potential for a substantial impact (on the result) of the failure to analyze participants in the group to which they were randomized? |  | No/ Probably No | Yes/ Probably Yes/ No information |
| **Bias due to missing outcome data** | | | | |
| 7 | Were data for this outcome available for all, or nearly all, participants randomized? | 1 | Yes/ Probably Yes | No/ Probably No/ No information |
|  | If No/ Probably No/ No Information to 7: Is there evidence that the result was not biased by missing outcome data? |  | Yes/ Probably Yes | No/ Probably No/ No information |
|  | If No/ Probably No to the question above: Could missingness in the outcome depend on its true value? |  | No/ Probably No | Yes/ Probably Yes/ No information |
|  | If Yes/ Probably Yes/ No Information to the question above: Is it likely that missingness in the outcome depended on its true value? |  | No/ Probably No | Yes/ Probably Yes/ No information |
| **Bias in measurement of the outcome** | | | | |
| 8 | Was the method of measuring the outcome inappropriate? | 1 | No/ Probably No | Yes/ Probably Yes/ No information |
| 9 | Could measurement or ascertainment of the outcome have differed between intervention groups? | 1 | No/ Probably No | Yes/ Probably Yes/ No information |
|  | If No/ Probably No/ No Information to 8 and 9: Were outcome assessors aware of the intervention received by study participants? |  | No/ Probably No | Yes/ Probably Yes/ No information |
|  | If Yes/ Probably Yes/ No Information to the question above: Could the assessment of the outcome have been influenced by knowledge of the intervention received? |  | No/ Probably No | Yes/ Probably Yes/ No information |
|  | If Yes/ Probably Yes/ No Information to the question above: Is it likely that assessment of the outcome was influenced by knowledge of intervention received? |  | No/ Probably No | Yes/ Probably Yes/ No information |
| **5. Bias in selection of the reported result** | | | | |
| 10 | 5.1 Were the data that produced this result analyzed in accordance with a prespecified analysis plan that was finalized before unblinded outcome data were available for analysis? | 1 | Yes/ Probably Yes | No/ Probably No/ No information |
|  | Is the numerical result being assessed likely to have been selected, on the basis of the results, from: |  |  |  |
| 11 | (1) multiple eligible outcome measurements (eg, scales, definitions, time points) within the outcome domain? | 1 | No/ Probably No | Yes/ Probably Yes/ No information |
| 12 | (2) multiple eligible analyses of the data? | 1 | No/ Probably No | Yes/ Probably Yes/ No information |
|  | Total | 12 |  |  |

For No. 4, 5, 6, 7, 8, 9, and 10 appraisal items, all criteria should be met to give the score.

12~10: low risk; 10~8: some concern; 8 and below: “high risk”.

## Table S4. Scoring system for interviews using CASP Checklist

| Appraisal items | | Maximum Score | Individual score | |
| --- | --- | --- | --- | --- |
|  |  |  | 1 | 0 |
| **Section A: Are the results valid?** | | | | |
| 1 | Was there a clear statement of the aims of the research? | 1 | Yes | No/ Can’t Tell |
| 2 | Is a qualitative methodology appropriate? | 1 | Yes | No/ Can’t Tell |
|  | Is it worth continuing? | | | |
| 3 | Was the research design appropriate to address the aims of the research? | 1 | Yes | No/ Can’t Tell |
| 4 | Was the recruitment strategy appropriate to the aims of the research? | 1 | Yes | No/ Can’t Tell |
| 5 | Was the data collected in a way that addressed the research issue? | 1 | Yes | No/ Can’t Tell |
| 6 | Has the relationship between researcher and participants been adequately considered? | 1 | Yes | No/ Can’t Tell |
| **Section B: What are the results?** | | | | |
| 7 | Have ethical issues been taken into consideration? | 1 | Yes | No/ Can’t Tell |
| 8 | Was the data analysis sufficiently rigorous? | 1 | Yes | No/ Can’t Tell |
| 9 | Is there a clear statement of findings? | 1 | Yes | No/ Can’t Tell |
| **Section C: Will the results help locally?** | | | | |
| 10 | How valuable is the research? | 1 | Yes | No/ Can’t Tell |
|  | Total | 10 |  |  |

10~7: low risk；7~5: some concerns; 5 and below: high risk.
