# Supplementary material for: The Prevalence, Features, Influencing Factors, and Solutions for COVID-19 Vaccine Misinformation: Systematic Review
Source: JMIR Public Health Surveill. 2023 Jan 11;9:e40201. doi: 10.2196/40201 (PMC9838721; doi:10.2196/40201)
Supplement: Multimedia Appendix 4 [file publichealth_v9i1e40201_app4.docx]

## Appendix4. Characteristics of included studies

| Reference | Study Design | Data source | Region | Country | Study period | Study Phase | Study population and sample size | Risk of bias |
| --- | --- | --- | --- | --- | --- | --- | --- | --- |
| Eshel et al., 2022 [89] | Cross-sectional study using online survey | Israeli Jewish adults | European | Israel | 8 Oct 2021 to 12 Oct 2021 | Phase 2 | 2,002 Israeli Jewish adults | Low risk |
| Andrade et al.,2021 [93] | Cross-sectional study using online survey | Venezuerla university student | American | Venezuela | 3weeks in Feb 2021 | Phase 2 | 273 Venezuerla university students | Low risk |
| Exline et al.,2022 [99] | Cross-sectional study using online survey | USA adults | American | USA | 28 Feb 2022 to 2 Mar 2022 | Phase 3 | 3,196 USA adults | Low risk |
| Garcia et al., 2021 [39] | Telephone interview using semi-structured questionnaire | Latino youth and parents | American | USA: Oregon | Jul 2020 to Jan 2021 | Phase 1 & 2 | 24 Latino youth and 22 parents (mother) | Low risk |
| Gesualdo et al.,2022 [40] | Internet-based study using qualitative text analysis | Social media: Twitter | European | Italy | 15 Nov 2019 to 22 Jun 2020 | Phase 1 | 2,463 vaccine-related tweets posted in Italy | Low risk |
| An et al., 2021 [83] | Internet-based study using manual classification | Google trends | American | USA | 1 Jan 2021 to 16 Mar 2021 | Phase 2 | Google trends data | Low risk |
| Chen et al., 2022 [84] | Internet-based study using natural language processing (NLP) method | Internet news media sources | Western Pacific | China (Taiwan) | 1 Mar 2021 to 25 Dec 2021 | Phase 2 & 3 | 791,183 COVID-19 and vaccine news items collected from 26 Internet news media sources | Low risk |
| Bishnoi et al., 2021 [35] | Focus group interview | Indian participants | South-East Asian | India: Rajasthan | 12 Apr 2021 to 17 Apr 2021 | Phase 2 | 35 Indian participants | High risk |
| Bíró‑Nagy and Szász, 2022 [32] | Cross-sectional study using in-person survey | Hungary adults | European | Hungary | 2 Mar 2021 to 11 Mar 2021 | Phase 2 | 1,000 Hungary adults | Low risk |
| Calac et al., 2022 [80] | Internet-based study using manual classification | Social media: Twitter | World-wide | NA | 31 Jan 2021 to 6 Feb 2021 | Phase 2 | 436 tweets with keywords "Hank Aaron" and "vaccine" | Low risk |
| Caycho-Rodríguez et al.,2022 [98] | Cross-sectional study using online survey | Adults in 13 Latin American countries | American | 13 Latin American countries: Argentina, Bolivia, Chile, Colombia, Cuba, Ecuador, El Salvador, Guatemala, Mexico, Paraguay, Peru, Uruguay and Venezuela | 15 Sep 2021 to 25 Oct 2021 | Phase 2 | 5,779 adults in 13 Latin American countries | Low risk |
| Buturoiu et al.,2021 [96] | Cross-sectional study using online survey | Romania adults | European | Romania | 1 Apr 2021 to 9 Apr 2021 | Phase 2 | 945 Romania adults | Low risk |
| Kulkarni et al., 2021 [47] | Cross-sectional study using online survey | Indian adults | South-East Asian | India | NA | NA | 216 Indian adults | Some concern |
| Jemielniak and Krempovych, 2021 [79] | Internet-based study using Min-Hash local-sensitive hashing method | Social media: Twitter | World-wide | NA | 1 Jan 2021 to 22 Mar 2021 | Phase 2 | 221,922 tweets containing "#AstraZeneca" | Low risk |
| Kumar et al., 2022 [109] | In-depth interview | Indian interviewees from a wide range of social background | South-East Asian | India: Chennai | Jan 2021 to Feb 2021 | Phase 2 | 18 Indian interviewees from a wide range of social background | Low risk |
| Lin et al., 2022 [100] | Cross-sectional study using online survey | Representative respondents from 6 countries | American, European, Western Pacific | 6 countries: China (Hong Kong), Japan, South Korea, Singapore, the UK, and the US | 15 Jun 2021 to 30 Jun 2021 | Phase 2 | 6,764 representative respondents from 6 countries | Low risk |
| Lanyi et al., 2021 [112] | Internet-based study using natural language processing (NLP) and manual classification | Social media: Twitter | European | UK: London | 30 Nov 2020 to 15 Aug 2021 | Phase 2 | 302 COVID-19 vaccine related tweets with negative sentiment located in London | Low risk |
| Kricorian et al., 2022 [31] | Cross-sectional study using online survey throught email and text message | USA adults who think vaccine not going to be safe | American | USA | 4 Jan 2021 to 10 Jan 2021 | Phase 2 | 1,950 USA adults who think vaccine not going to be safe | Low risk |
| Lurie et al., 2022 [54] | Internet-based study using manual classification | Database of online article | World-wide | NA | 27 Jul 2020 to 30 Jun 2021 | Phase 1 & 2 | 1,298,054 English-language articles on COVID-19 vaccines | Low risk |
| Kant, et al., 2021 [45] | Internet-based study using manual classification | Social meida: Facebook | Western Pacific | Fiji | NA | NA | 387 Facebook posts in Fiji | Some concern |
| Marchlewska et al., 2022 [105] | Cross-sectional study using online survey | Polish adults | European | Poland | Mar 2020 and Dec 2020 | Phase 1 & 2 | 1,239 Polish adults | Low risk |
| Mahmud et al., 2021 [95] | Cross-sectional study using online survey | Bangladeshi adults | South-East Asian | Bangladesh | Feb 2021 to Mar 2021 | Phase 2 | 403 Bangladeshi adults | Low risk |
| Lamptey et al., 2022 [50] | Cross-sectional study using online survey | Respondents in Africa | African | Africa | 5 Jan 2021 to 5 Jul 2021 | Phase 2 | 2,500 respondents in Africa | Low risk |
| Magee et al., 2022 [77] | Interview using semi-structured questionnaire | Patients belonging to ethnic groups | European | UK: London | May 2021 and Aug 2021 | Phase 2 | 38 patients belonging to ethnic groups | Low risk |
| Lee et al., 2022 [51] | Cross-sectional study using open-ended survey | Full-time working professionals in the USA | American | USA | Mar 2021 | Phase 2 | 505 full-time working professionals in the USA | Low risk |
| Liao et al., 2022[53] | Internet-based study using inductive method for creating photographic typologies | Google image | American | USA | 4 Nov 2021 to 13 Jan 2022 | Phase 2 & 3 | 150 public protest slogans in response to the Biden Administration’s announcement of two COVID-19 vaccination regulations | Low risk |
| Laforet et al., 2022[49] | Internet-based study using manual classification | Social media: Youtube | World-wide | NA | Before 17 Feb 2021 | Phase 1 & 2 | 50 most viewed English videos on pregnancy and COVID-19 vaccination | Low risk |
| Li et al., 2022[52] | Internet-based study using manual classification | Social media: Youtube | World-wide | NA | Before 21 Jul 2021 | Phase 1 & 2 | 150 most-viewed videos related COVID-19 vaccine | Low risk |
| Savolainen, 2021[61] | Internet-based study using manual classification | Social media: Reddit | World-wide | NA | 1 Jan 2021 to 7 Feb 2021 | Phase 2 | 1,877 messages from Reddit | High risk |
| Saini et al., 2022 [111] | Internet-based study using multiple methods | Social media: Twitter | American | USA | 26 Apr 2021 to 26 Aug 2021 | Phase 2 | 8,556 antivaccine English-language tweets from the United States | Low risk |
| Oleksy et al., 2022[59] | Follow-up study | Polish people | European | Poland | 4 May 2020 to 7 May 2020 in study 1; 4 Jun 2020 to 17 Jun 2020 in study 2; 7 Jul 2020 to 17 Jul 2020 in study 3; 3 Dec 2020 to 12 Dec 2020 in study 4 | Phase 1 | 1,130 Polish people | Some concern |
| Perlis et al., 2022[104] | Follow-up study using online survey | USA adults | American | USA | 1 Apr 2021 to 3 May 2021 in suvrey 1, 9 Jun 2021 to 7 Jul 2021 in survey 2 | Phase 2 | 15,464 USA adults in survey 1;  2,809 in survey 2 | Low risk |
| Savoia e al., 2022 [86] | Cross-sectional study using online survey | Adults from the USA, Canada, and Italy | American, European | US, Canada, Italy | 21 May 2021 to 28 May 2021 | Phase 2 | 2,697 adults from the USA, Canada, and Italy | Low risk |
| Okoro et al., 2021[58] | mixed-methods: Cross-sectional study and interview | Black Communities in US | American | USA | 24 Aug 2020 to 28 Nov 2020 | Phase 1 | 183 Black Communities respondents in US | Some concern |
| Ngai et al., 2022 [72] | Internet-based study using manual analysis | Multiple: Facebook, Instagram and Twitter | World-wide | NA | 15 Sep 2019 to 16 Aug 2020 | Phase 1 | 350 online posts containing misinformation on Facebook, Instagram and Twitter | Low risk |
| Obreja et al., 2022[57] | Internet-based study using thematic analysis | Social media: Facebook | European | Romania | 26 Sep 2021 to 10 Oct 2021 | Phase 2 | 137 comments post on Romanian offcial Facebook page highlighted anti-vaccination narratives | Low risk |
| Neely et al., 2021[56] | Cross-sectional study using online survey | Florida adults | American | USA: Florida | 3 Jun 2021 to 14 Jun 2021 | Phase 2 | 600 Florida adults | Low risk |
| Wawrzuta et al., 2022 [115] | Internet-based study using manually coding | Social media: Twitter | European | Poland | 1 Aug 2021 to 1 Feb 2022 | Phase 2 & 3 | 200 most popular posts with hashtag “#szczepimysie” (“#we vaccinate ourselves”) | Low risk |
| Wonodi et al., 2022 [66] | Focus group and key informant interview | Nigeria adults | African | Nigeria | 1 Feb 2021 to 8 Feb 2021 | Phase 2 | 178 Nigeria adults | Low risk |
| Wong et al., 2021 [28] | Internet-based study using manual classification | Multiple social media: Instagram and Facebook | World-wide | NA | 16 Nov 2020 to 18 Nov 2020 | Phase 1 | 9,705 Instagram and Facebook English comments on BBC World News and CNN International | Low risk |
| Abbas et al., 2022 [70] | Cross-sectional study using survey | Pakistan respondents >15 years | Eastern Mediterranean | Pakistan: Rawalpindi | Feb 2021 to Jun 2021 | Phase 2 | 100 Pakistan respondents >15 years | High risk |
| Ginossar et al., 2022 [123] | Internet-based study using LDA | Social media: Twitter | World-wide | NA | 1 Feb 2020 to 23 Jun 2020 | Phase 1 | 2,097 English tweets with Youtube video links | Low risk |
| Al-Rawi et al., 2022 [71] | Internet-based study using manual classification | Social media: Twitter | African | Arabic area | 1 Aug 2006 to 10 Oct 2021 | Phase 1 & 2 | 476,048 tweets in Arabic | Low risk |
| Yousuf et al., 2021 [30] | Cross-sectional study using survey throught natioanl newspaper | Dutch vaccine hesitant people | European | Dutch | Dec 2020 | Phase 1 | 24,722 Dutch vaccine hesitant people | Some concern |
| Yang et al., 2021 [68] | Cross-sectional study using online survey | Chinese adults | Western Pacific | China | 1 Apr 2021 to 8 Apr 2021 | Phase 2 | 1,890 Chinese adults | Low risk |
| Wang et al., 2022 [63] | Face-to-face and online interviews | Adults from 4 countries | American, European, Western Pacific | 4 countries: China (Taiwan), USA, Netherlands, and Haiti | Nov 2020 to Mar 2021 | Phase 1 & 2 | 47 adults from 4 countries | Low risk |
| Yaseen et al., 2022 [69] | In-depth interview using semi-structured questionnaire | Pakistani pharmacists | Eastern Mediterranean | Pakistan | NA | NA | 36 Pakistani pharmacists | Low risk |
| Watermeyer et al., 2022 [64] | Interview | Young community healthcare workers in Soweto, South Africa | African | South Africa | Sep 2021 to Nov 2021 | Phase 2 | 20 young community healthcare workers in Soweto, South Africa | Low risk |
| Jiang et al.,2021 [81] | Internet-based study using LDA | Social media: Twitter | American | USA | 21 Feb 2020 to 20 Mar 2020 | Phase 1 | 100,209 tweets related to coronavirus and vaccines | Low risk |
| Hammad et al., 2022 [88] | Cross-sectional study using online survey | Jodan respondents | Eastern Mediterranean | Jordan | May 2021 and Aug 2021 | Phase 2 | 1,195 Jodan respondents | Low risk |
| Criss et al., 2021 [122] | Internet-based study using manual classification | Social media: Twitter | American | USA | Oct 2020 to Jan 2021 | Phase 1 & 2 | 1,110 tweets related to COVID-19 vaccines | Low risk |
| Herrera-Peco et al., 2021[15] | Internet-based study using manual classification | Social media: Twitter | World-wide | NA | 14 Dec 2020 and 28 Dec 2020 | Phase 2 | 12,340 Spanish Twitter interactions with hastag #yonomevacuno (#idonotgetvaccinated) | Low risk |
| Griffith et al., 2021[41] | Internet-based study using manual classification | Social media: Twitter | American | Canada | 10 Dec 2020 to 23 Dec 2020 | Phase 2 | 605 tweets about COVID-19 vaccine hesitancy | Low risk |
| Islam et al., 2021 [85] | Internet-based study using manual classification | Multiple: Google, Google Fact Check, Facebook, YouTube, Twitter, fact-checking agency websites, and websites of television and newspaper | World-wide | 52 countries | 31 Dec 2019 to 30 Nov 2020 | Phase 1 | 637 online reports about rumors and conspiracy theories | Low risk |
| Hughes et al., 2021 [73] | Internet-based study using manual classification | Multiple social media: YouTube, Twitter, Facebook, and Instagram | World-wide | NA | NA | NA | Online post about anti-vaccine content and/or COVID denialism | Low risk |
| Larrondo-Ureta et al., 2021 [113] | Internet-based study | Social media: Twitter | World-wide | NA | 14 Dec 2020 to 7 Feb 2021 | Phase 2 | 62,045 tweets with hashtag #yonomevacuno  ("against vaccination"): | Low risk |
| Kalichman et al, 2021 [114] | Internet-based study | Social media: Facebook | World-wide | NA | 1 Feb 2020 to 31 May 2020 | Phase 1 | 2,060 Facebook posts from four antivaccine groups | Low risk |
| Hernández-García et al., 2021[43] | Internet-based study using manual classification | Social media: YouTube | World-wide | NA | 18 Mar 2020 to 5 Feb 2021 | Phase 1 & 2 | 118 Spanish YouTube videos | Low risk |
| Jennings et al., 2021 [108] | Focus group interview | UK adults | European | UK | 30 Nov 2020 to 7 Dec 2020 | Phase 1 | 5 focus groups with 29 UK participants and 1,476 adults in the UK | Low risk |
| Faezi et al., 2021 [101] | Cross-sectional study using online survey | Participants from 42 nations | World-wide | 42 African and Middle East countries | 15 Feb 2021 to 15 Apr 2021 | Phase 2 | 1,880 individuals from 42 different countries | Low risk |
| Ebrahimi et al., 2021 [38] | Cross-sectional study using online survey | Norwegian adults | European | Norway | 23 Jan 2021 to 2 Feb 2021 | Phase 2 | 4,571 Norwegian adults | Low risk |
| Hernandez et al., 2021 [78] | Internet-based study | Social media: Twitter | World-wide | NA | Jul 2020 | Phase 1 | Over 1 million tweets | Low risk |
| Chan et al., 2021 [121] | Internet-based study using manual classification | Social media: YouTube | World-wide | NA | Before 10 Dec 2020 | Phase 1 | 48 COVID-19 most viewed YouTube videos | Low risk |
| Hornsey et al., 2021 [90] | Cross-sectional study using online survey | Participants from eight nations | American, European, Western Pacific | 8 countries: Australia, France, Germany, Italy, the Netherlands, Spain, UK, USA | 17 Mar 2020 to 2 Jul 2020 | Phase 1 | 4,245 participants from 8 countries | Low risk |
| Jensen et al., 2021 [97] | Cross-sectional study using postcard survey | Germany residents | European | Germany | 30 Oct 2020 to 14 Dec 2020 | Phase 1 | 725 participants | Low risk |
| Aloweidi et al., 2021 [102] | Cross-sectional study using online survey | Medical personnel and non-medical adults | Eastern Mediterranean | Jordan: Amman | 22 Jan 2021 to 28 Feb 2021 | Phase 2 | 287 medical personnel and 359 non-medical adults | Low risk |
| Arshad et al., 2021 [74] | Cross-sectional study using online survey | Pakistan residents | Eastern Mediterranean | Pakistan | Jan 2021 | Phase 2 | 2,158 Pakistan adults | Low risk |
| Alliheibi et al., 2021 [33] | Internet-based study | Social media: Twitter | Eastern Mediterranean | Saudi Arabia | 15 Dec 2020 to 25 May 2021 | Phase 1 | 37,467 tweets | Some concern |
| Baines et al., 2021 [110] | Internet-based study using manual classification | Social media: Parler | American | USA | 20 Nov 2020 to 6 Jan 2021 | Phase 1 & 2 | 400 Parler posts of two hashtags (#COVID19Vaccine and #NoCovidVaccine). | Low risk |
| Basch et al., 2020 [120] | Internet-based study using manual classification | Social media: YouTube | World-wide | NA | 6 Apr 2020 | Phase 1 | 100 widely viewed YouTube videos | Low risk |
| Basch et al., 2021 [34] | Internet-based study using manual classification | Social media: TikTok | World-wide | NA | NA | NA | 100 trending TikTok videos | Low risk |
| Sallam et al., 2021 [92] | Cross-sectional study using online survey | Residents of Jordan, Kuwait and Saudi Arabia | Eastern Mediterranean | 3 Arab countries: Jordan, Kuwait and other Arab countries | 14 Dec 2020 to 18 Dec 2020 | Phase 2 | 3,414 Arabic residents aged 16 years and above | Low risk |
| Ruiz and Bell, 2021 [82] | Cross-sectional study using online survey | USA English-speaking adults | American | USA | 15 Jun to 16 Jun 2020 | Phase 1 | 804 adult USA residents | Low risk |
| Romer and Jamieson, 2020 [107] | Panel study using survey | USA adults | American | USA | 17 Mar and 27 Mar 2020 (Wave 1), 10 Jul 2020and 21 Jul 2020 (Wave 2) | Phase 1 | 1,050 USA residents in wave 1; 840 residents in wave2 | Low risk |
| Thelwall et al., 2021 [62] | Internet-based study using manual classification | Social media: Twitter | World-wide | NA | 10 Mar 2020 and 5 Dec 2020 | Phase 1 | 446 tweets about COVID-19 vaccine hesitancy | Low risk |
| Sallam et al., 2021 [60] | Cross-sectional study using online survey | University students in Jordan | Eastern Mediterranean | Jordan | 19 Jan 2020 to 23 Jan 2020 | Phase 1 | 1,106 university students in Jordan | Low risk |
| Romer and Jamieson, 2021 [1] | Panel study using survey | USA adults | American | USA | Mar 2020 and Jul 2020 | Phase 1 | 840 USA residents | Low risk |
| Roozenbeek et al., 2020 [117] | Cross-sectional study using online survey | Participants from five countries | American, European | 4 countries: Ireland, USA, Spain, Mexico, UK | Apr 2020 and May 2020 | Phase 1 | 5,000 participants from 5 countries | Low risk |
| Wawrzuta et al., 2021 [65] | Internet-based study using machine learning | Social media: Facebook | European | Poland | Sep 2020 to Dec 2020 | Phase 1 | 3,414 Facebook comments | Low risk |
| Hossain et al., 2021 [44] | Cross-sectional study using online survey | Bangladesh residents | South-East Asian | Bangladesh | 1 Feb 2021 to 7 Feb 2021 | Phase 2 | 1497 adult Bangladesh residents | Low risk |
| Magadmi and Kamel, 2021 [29] | Cross-sectional study using online survey | Saudi Arabia residents | Eastern Mediterranean | Saudi Arabia | May 2020 | Phase 1 | 3,101 Saudi Arabia adults | Low risk |
| Solís Arce et al., 2021 [76] | Cross-sectional study using online survey | Participants from 13 countries | World-wide | 13 countries: Russia, USA and 10 low- and middleincome countries (LMICs) in Asia, Africa, and South America; | Jun 2020 and Jan 2021 | Phase 1 & 2 | 44,260 respondents from 13 countries | Low risk |
| Wu et al., 2021 [67] | Internet-based study using LDA (topic modeling method) and LIWC (capture psychological information) | Social media: Reddit | World-wide | NA | 1 March 2020 to 15 Dec 2020 | Phase 1 | 172,091 reddit comments | Low risk |
| Küçükali et al., 2021 [46] | Internet-based study using manual classification | Social media: Twitter | European | Turkey | 9 Dec 2020 to 8 Jan 2021 | Phase 2 | 1,041 Turkish tweets | Low risk |
| Hamdan et al., 2021 [42] | Cross-sectional study using online survey | Students of the American University of Beirut | Eastern Mediterranean | Lebanon: American University of Beirut (AUB) | 11 May 2021 to 18 Jun 2021 | Phase 2 | 800 undergraduate and graduate students in Lebanon | Low risk |
| Dereje et al., 2021 [37] | Multiple: cross-sectioanl study using face-to-face survey and in-depth interview | Adults in Ethiopia, Addis Ababa, Akaki Kality sub-city | African | Ethiopia: Addis Ababa, Akaki Kality sub-city | 20 Jan 2021 to 31 Jan 2021 | Phase 2 | 409 Ethiopia adults for quantitative study, 24 participants for qualitative study | Low risk |
| Muric et al., 2021[55] | Internet-based study | Social media: Twitter | World-wide | NA | 18 Oct 2020 to 21 Apr 2021 | Phase 1 & 2 | 1.8 million tweets indicating opposition to vaccines | Low risk |
| Kumar et al., 2021[48] | Internet-based study using Structural Topic modeling | Social media: Reddit | World-wide | NA | 1 Jan 2020 to 14 Dec 2020 | Phase 1 | 266,840 Reddit posts | Low risk |
| Charquero-Ballester et al., 2021[36] | Internet-based study using manual classification | Social media: Twitter | World-wide | NA | 1 Mar 2021 to 31 Mar 2021 | Phase 2 | 2,029 tweets about the COVID-19 vaccine misinformation | Low risk |
| Manby et al., 2021 [94] | In-depth interview | Health care workers in UK | European | UK: London | 16 Dec 2020 to 3 Mar 2021 | Phase 2 | 24 health care workers in London | Low risk |
| Pierri et al., 2021 [103] | Ecological study | Social media: Twitter | American | USA | 4 Jan 2021 to 25 Mar 2021 | Phase 2 | 55 million tweets from the CoVaxxy dataset, and daily vaccination rates recorded by US CDC | Low risk |
| Sharevski et al., 2021 [104] | Randomized controlled trial measured by post-exposure survey | USA adult Twitter users | American | US | Jan 2021 and Feb 2021 | Phase 2 | 319 USA Twitter users | High risk |
| Jin et al., 2021 [116] | Randomized controlled trial measured by pre- and post-exposure self-reported surveys | Pakistani adults | Eastern Mediterranean | Pakistan | NA | NA | 320 Pakistani adults | Some concern |
| Loomba et al., 2021 [91] | Randomized controlled trial measured by pre- and post-exposure self-reported surveys | UK and USA residents | American, European | USA, UK | 7 Sep 2020 to 14 Sep 2020 | Phase 1 | 8,001 nationally representative samples from UK and USA | Some concern |
| Yousuf et al., 2021 [124] | Randomized controlled trial measured by pre- and post-exposure self-reported surveys | Viewers of Dutch Television | European | Netherland | 13 Oct 2020 to 24 Oct 2020 | Phase 1 | 475 participants for the nondebunking group, and 505 participants for the debunking group | Low risk |
| Dai et al.,2022 [128] | Randomized controlled trial using 2 by 3 desgin | USA adults | American | USA | NA | NA | 634 USA adults | Low risk |
| Lu and Zhong, 2022 [125] | Randomized controlled trial using 3 phase between-subject survey | Participants from Amazon’s Mechanical Turk | American | USA | late Feb 2021 | Phase 2 | 363 participants from Amazon’s Mechanical Turk | Low risk |
| Helfers et al., 2022 [118] | Randomized controlled trial using pre- and post- online survey | German participants | European | Germany | Mar 2021 | Phase 2 | 588 German participants | Low risk |
| Piltch-Loeb et al., 2022 [127] | Quasi experiments using between-group survey | Unvaccinated US adults | American | USA | 3 Jun 2021 to 5 Jun 2021 | Phase 2 | 1,991 unvaccinated US adults | Low risk |
| Talabi et al., 2022 [126] | Two quasi experiments using pre- and post- online survey | Social media users in Nigeria | African | Nigeria | NA | NA | 470 social media users in Nigeria | Low risk |
| Thaker and Subramanian, 2021 [75] | Randomized controlled trial using pre- and post- online survey | New Zealand adults | Western Pacific | New Zealand | 15 Feb 2021 to 6 Mar 2021 | Phase 2 | 1,083 New Zealand adults | Low risk |
| Vivion et al., 2022 [119] | Randomized controlled trial using pre- and post- online survey | Canadians aged 50 years and older | American | Canada | 8 Mar 2021 to 17 Mar 2021 | Phase 2 | 2,500 Canadians aged 50 years and older | Low risk |

# References:

1. Romer D, Jamieson KH. Patterns of Media Use, Strength of Belief in COVID-19 Conspiracy Theories, and the Prevention of COVID-19 From March to July 2020 in the United States: Survey Study. J Med Internet Res 2021 Apr 27;23:e25215 [doi: 10.2196/25215] [Medline: 33857008]

15. Herrera-Peco I, Jiménez-Gómez B, Romero Magdalena CS, Deudero JJ, García-Puente M, Benítez De Gracia E, et al. Antivaccine Movement and COVID-19 Negationism: A Content Analysis of Spanish-Written Messages on Twitter. Vaccines (Basel) 2021 Jun 15;9:656 [doi: 10.3390/vaccines9060656] [Medline: 34203946]

28. Wong LP, Lin Y, Alias H, Bakar SA, Zhao Q, Hu Z. COVID-19 Anti-Vaccine Sentiments: Analyses of Comments from Social Media. Healthcare (Basel) 2021 Dec 09;9:1530 [doi: 10.3390/healthcare9111530] [Medline: 34828576]

29. Magadmi RM, Kamel FO. Beliefs and barriers associated with COVID-19 vaccination among the general population in Saudi Arabia. BMC Public Health 2021 Jul 21;21:1438. [doi: 10.1186/s12889-021-11501-5] [Medline: 34289817]

30. Yousuf H, van der Linden S, van Essen T, Gommers D, Scherder E, Narula J, et al. Dutch Perspectives Toward Governmental Trust, Vaccination, Myths, and Knowledge About Vaccines and COVID-19. JAMA Netw Open 2021 Dec 01;4:e2140529 [doi: 10.1001/jamanetworkopen.2021.40529] [Medline: 34967887]

31. Kricorian K, Civen R, Equils O. COVID-19 vaccine hesitancy: misinformation and perceptions of vaccine safety. Hum Vaccin Immunother 2022 Dec 31;18:1950504 [doi: 10.1080/21645515.2021.1950504] [Medline: 34325612]

32. Bíró-Nagy A, Szászi áJ. The roots of COVID-19 vaccine hesitancy: evidence from Hungary. J Behav Med 2022 May 14:1-16 [doi: 10.1007/s10865-022-00314-5] [Medline: 35567729]

33. Alliheibi FM, Omar A, Al-Horais N. Opinion Mining of Saudi Responses to COVID-19 Vaccines on Twitter: A Computational Linguistic Approach. International Journal of Advanced Computer Science and Applications 2021;12:72-78. [doi: 10.14569/IJACSA.2021.0120610]

34. Basch CH, Meleo-Erwin Z, Fera J, Jaime C, Basch CE. A global pandemic in the time of viral memes: COVID-19 vaccine misinformation and disinformation on TikTok. Hum Vaccin Immunother 2021 Aug 03;17:2373-2377 [doi: 10.1080/21645515.2021.1894896] [Medline: 33764283]

35. Bishnoi HR, Avasthi RD, Sharma SK. A Qualitative Study To Myths And Reality About Covid-19 Vaccination Among People In Western Rajasthan. Journal of Cardiovascular Disease Research 2022 Apr;12:476-482

36. Charquero-Ballester M, Walter J, Nissen I, Bechmann A. Different types of COVID-19 misinformation have different emotional valence on Twitter. Big Data & Society 2021 Sep 22;8:205395172110412. [doi: 10.1177/20539517211041279]

37. Dereje N, Tesfaye A, Tamene B, Alemeshet D, Abe H, Tesfa N, et al. COVID-19 vaccine hesitancy in Addis Ababa, Ethiopia: a mixed-method study. BMJ Open 2022 May 30;12:e052432 [doi: 10.1136/bmjopen-2021-052432] [Medline: 35636790]

38. Ebrahimi OV, Johnson MS, Ebling S, Amundsen OM, Halsøy Ø, Hoffart A, et al. Risk, Trust, and Flawed Assumptions: Vaccine Hesitancy During the COVID-19 Pandemic. Front Public Health 2021;9:700213 [doi: 10.3389/fpubh.2021.700213] [Medline: 34277557]

39. Garcia J, Vargas N, de la Torre C, Magana Alvarez M, Clark JL. Engaging Latino Families About COVID-19 Vaccines: A Qualitative Study Conducted in Oregon, USA. Health Educ Behav 2021 Dec;48:747-757 [doi: 10.1177/10901981211045937] [Medline: 34596462]

40. Gesualdo F, Parisi L, Croci I, Comunello F, Parente A, Russo L, et al. How the Italian Twitter Conversation on Vaccines Changed During the First Phase of the Pandemic: A Mixed-Method Analysis. Front Public Health 2022;10:824465 [doi: 10.3389/fpubh.2022.824465] [Medline: 35664110]

41. Griffith J, Marani H, Monkman H. COVID-19 Vaccine Hesitancy in Canada: Content Analysis of Tweets Using the Theoretical Domains Framework. J Med Internet Res 2021 Apr 13;23:e26874 [doi: 10.2196/26874] [Medline: 33769946]

42. Bou Hamdan M, Singh S, Polavarapu M, Jordan T, Melhem N. COVID-19 vaccine hesitancy among university students in Lebanon. Epidemiol. Infect 2021 Nov 02;149:e242. [doi: 10.1017/s0950268821002314]

43. Hernández-García I, Gascón-Giménez I, Gascón-Giménez A, Giménez-Júlvez T. Information in Spanish on YouTube about Covid-19 vaccines. Hum Vaccin Immunother 2021 Nov 02;17:3916-3921 [doi: 10.1080/21645515.2021.1957416] [Medline: 34375570]

44. Hossain MB, Alam MZ, Islam MS, Sultan S, Faysal MM, Rima S, et al. COVID-19 vaccine hesitancy among the adult population in Bangladesh: A nationwide cross-sectional survey. PLoS One 2021;16:e0260821. [doi: 10.1371/journal.pone.0260821] [Medline: 34882726]

45. Kant R, Varea RR, Titifanue J. COVID-19 vaccine online misinformation in Fiji: Preliminary findings. Pacific Journalism Review 2022;27:47-62 [doi: 10.24135/pjr.v27i1&2.1189]

46. Kükali H, Ata? Ö, Palteki AS, Toka? AZ, Hayran O. Vaccine Hesitancy and Anti-Vaccination Attitudes during the Start of COVID-19 Vaccination Program: A Content Analysis on Twitter Data. Vaccines (Basel) 2022 Jan 21;10:161 [doi: 10.3390/vaccines10020161] [Medline: 35214620]

47. Kulkarni M, Khurana K. Acceptance and myths regarding covid vaccination among general population. Medical Science 2022;26:ms255e2245 [doi: 10.54905/disssi/v26i124/ms255e2245]

48. Kumar N, Corpus I, Hans M, Harle N, Yang N, McDonald C, et al. COVID-19 vaccine perceptions in the initial phases of US vaccine roll-out: an observational study on reddit. BMC Public Health 2022 Mar 07;22:446 [doi: 10.1186/s12889-022-12824-7] [Medline: 35255881]

49. Laforet PE, Basch CH, Tang H. Understanding the content of COVID-19 vaccination and pregnancy videos on YouTube: An analysis of videos published at the start of the vaccine rollout. Hum Vaccin Immunother 2022 Nov 30;18:2066935 [doi: 10.1080/21645515.2022.2066935] [Medline: 35507867]

50. Lamptey E, Senkyire EK, Dorcas S, Benita DA, Boakye EO, Ikome T, et al. Exploring the myths surrounding the COVID-19 vaccines in Africa: the study to investigate their impacts on acceptance using online survey and social media. Clin Exp Vaccine Res 2022 May;11:193-208 [doi: 10.7774/cevr.2022.11.2.193] [Medline: 35799880]

51. Lee SK, Sun J, Jang S, Connelly S. Misinformation of COVID-19 vaccines and vaccine hesitancy. Sci Rep 2022 Aug 11;12:13681 [doi: 10.1038/s41598-022-17430-6] [Medline: 35953500]

52. Li HOY, Pastukhova E, Brandts-Longtin O, Tan MG, Kirchhof MG. YouTube as a source of misinformation on COVID-19 vaccination: a systematic analysis. BMJ Glob Health 2022 Mar;7:e008334 [doi: 10.1136/bmjgh-2021-008334] [Medline: 35264318]

53. Liao TF. Understanding Anti-COVID-19 Vaccination Protest Slogans in the US. Front. Commun 2022 Jun 30;7:941872 [doi: 10.3389/fcomm.2022.941872]

54. Lurie P, Adams J, Lynas M, Stockert K, Carlyle RC, Pisani A, et al. COVID-19 vaccine misinformation in English-language news media: retrospective cohort study. BMJ Open 2022 Jun 01;12:e058956 [doi: 10.1136/bmjopen-2021-058956] [Medline: 35649595]

55. Muric G, Wu Y, Ferrara E. COVID-19 Vaccine Hesitancy on Social Media: Building a Public Twitter Data Set of Antivaccine Content, Vaccine Misinformation, and Conspiracies. JMIR Public Health Surveill 2021 Nov 17;7:e30642 [doi: 10.2196/30642] [Medline: 34653016]

56. Neely SR, Eldredge C, Ersing R, Remington C. Vaccine Hesitancy and Exposure to Misinformation: a Survey Analysis. J Gen Intern Med 2022 Jan;37:179-187 [doi: 10.1007/s11606-021-07171-z] [Medline: 34671900]

57. Obreja DM. Narrative communication regarding the Covid-19 vaccine: a thematic analysis of comments on Romanian official Facebook page "RO Vaccinare". SN Soc Sci 2022;2:119 [doi: 10.1007/s43545-022-00427-3] [Medline: 35875608]

58. Okoro O, Kennedy J, Simmons G, Vosen EC, Allen K, Singer D, et al. Exploring the Scope and Dimensions of Vaccine Hesitancy and Resistance to Enhance COVID-19 Vaccination in Black Communities. J Racial Ethn Health Disparities 2022 Dec;9:2117-2130 [doi: 10.1007/s40615-021-01150-0] [Medline: 34553340]

59. Oleksy T, Wnuk A, Gambin M, Łyś A, Bargiel-Matusiewicz K, Pisula E. Barriers and facilitators of willingness to vaccinate against COVID-19: Role of prosociality, authoritarianism and conspiracy mentality. A four-wave longitudinal study. Pers Individ Dif 2022 May;190:111524 [doi: 10.1016/j.paid.2022.111524] [Medline: 35068638]

60. Sallam M, Dababseh D, Eid H, Hasan H, Taim D, Al-Mahzoum K, et al. Low COVID-19 Vaccine Acceptance Is Correlated with Conspiracy Beliefs among University Students in Jordan. Int J Environ Res Public Health 2021 Mar 01;18:2407 [doi: 10.3390/ijerph18052407] [Medline: 33804558]

61. Savolainen R. Assessing the credibility of COVID-19 vaccine mis/disinformation in online discussion. Journal of Information Science 2021 Aug 19:016555152110406. [doi: 10.1177/01655515211040653]

62. Thelwall M, Kousha K, Thelwall S. Covid-19 vaccine hesitancy on English-language Twitter. El Profesional de la Información 2021;30:1-13. [doi: 10.3145/epi.2021.mar.12]

63. Wang CW, de Jong EP, Faure JA, Ellington JL, Chen CHS, Chan CC. A matter of trust: a qualitative comparison of the determinants of COVID-19 vaccine hesitancy in Taiwan, the United States, the Netherlands, and Haiti. Hum Vaccin Immunother 2022 Nov 30;18:2050121 [doi: 10.1080/21645515.2022.2050121] [Medline: 35349382]

64. Watermeyer J, Scott M, Kapueja L, Ware LJ. To trust or not to trust: an exploratory qualitative study of personal and community perceptions of vaccines amongst a group of young community healthcare workers in Soweto, South Africa. Health Policy Plan 2022 Oct 12;37:1167-1176 [doi: 10.1093/heapol/czac060] [Medline: 35880606]

65. Wawrzuta D, Jaworski M, Gotlib J, Panczyk M. What Arguments against COVID-19 Vaccines Run on Facebook in Poland: Content Analysis of Comments. Vaccines (Basel) 2021 May 10;9:481 [doi: 10.3390/vaccines9050481] [Medline: 34068500]

66. Wonodi C, Obi-Jeff C, Adewumi F, Keluo-Udeke SC, Gur-Arie R, Krubiner C, et al. Conspiracy theories and misinformation about COVID-19 in Nigeria: Implications for vaccine demand generation communications. Vaccine 2022 Mar 18;40:2114-2121 [doi: 10.1016/j.vaccine.2022.02.005] [Medline: 35153088]

67. Wu W, Lyu H, Luo J. Characterizing Discourse about COVID-19 Vaccines: A Reddit Version of the Pandemic Story. Health Data Sci 2021;2021:9837856 [doi: 10.34133/2021/9837856] [Medline: 36405359]

68. Yang Z, Luo X, Jia H. Is It All a Conspiracy? Conspiracy Theories and People's Attitude to COVID-19 Vaccination. Vaccines (Basel) 2021 Sep 22;9:1051 [doi: 10.3390/vaccines9101051] [Medline: 34696159]

69. Yaseen MO, Saif A, Khan TM, Yaseen M, Saif A, Bukhsh A, et al. A qualitative insight into the perceptions and COVID-19 vaccine hesitancy among Pakistani pharmacists. Hum Vaccin Immunother 2022 Dec 31;18:2031455 [doi: 10.1080/21645515.2022.2031455] [Medline: 35192781]

70. Abbas SW, Zareen SF, Nisar S, Farooq A, Rasheed A, Saleem MU. COVID-19 Vaccines: Community Myths Vs Facts. Pakistan Armed Forces Medical Journal. 2022;72(2):497-500 [doi: 10.51253/pafmj.v72i2.6970] [Medline: 157247889]

71. Al-Rawi A, Fakida A, Grounds K. Investigation of COVID-19 Misinformation in Arabic on Twitter: Content Analysis. JMIR Infodemiology. 2022 Jul-Dec;2(2):e37007 [doi: 10.2196/37007] [Medline: 35915823]

72. Ngai CSB, Singh RG, Yao L. Impact of COVID-19 Vaccine Misinformation on Social Media Virality: Content Analysis of Message Themes and Writing Strategies. J Med Internet Res 2022 Jul 06;24:e37806. [doi: 10.2196/37806] [Medline: 35731969]

73. Hughes B, Miller-Idriss C, Piltch-Loeb R, Goldberg B, White K, Criezis M, et al. Development of a Codebook of Online Anti-Vaccination Rhetoric to Manage COVID-19 Vaccine Misinformation. Int J Environ Res Public Health 2021 Jul 15;18:7556 [doi: 10.3390/ijerph18147556] [Medline: 34300005]

74. Arshad MS, Hussain I, Mahmood T, Hayat K, Majeed A, Imran I, et al. A National Survey to Assess the COVID-19 Vaccine-Related Conspiracy Beliefs, Acceptability, Preference, and Willingness to Pay among the General Population of Pakistan. Vaccines (Basel) 2021 Jul 01;9:720 [doi: 10.3390/vaccines9070720] [Medline: 34358136]

75. Thaker J, Subramanian A. Exposure to COVID-19 Vaccine Hesitancy Is as Impactful as Vaccine Misinformation in Inducing a Decline in Vaccination Intentions in New Zealand: Results from Pre-Post Between-Groups Randomized Block Experiment. Front. Commun 2021 Aug 19;6:721982. [doi: 10.3389/fcomm.2021.721982]

76. Solís Arce JS, Warren SS, Meriggi NF, Scacco A, McMurry N, Voors M, et al. COVID-19 vaccine acceptance and hesitancy in low- and middle-income countries. Nat Med 2021 Aug;27:1385-1394 [doi: 10.1038/s41591-021-01454-y] [Medline: 34272499]

77. Magee L, Knights F, Mckechnie DGJ, Al-Bedaery R, Razai MS. Facilitators and barriers to COVID-19 vaccination uptake among ethnic minorities: A qualitative study in primary care. PLoS One 2022;17:e0270504 [doi: 10.1371/journal.pone.0270504] [Medline: 35802738]

78. Hernandez RG, Hagen L, Walker K, O'Leary H, Lengacher C. The COVID-19 vaccine social media : healthcare providers' missed dose in addressing misinformation and vaccine hesitancy. Hum Vaccin Immunother 2021 Sep 02;17:2962-2964 [doi: 10.1080/21645515.2021.1912551] [Medline: 33890838]

79. Jemielniak D, Krempovych Y. An analysis of AstraZeneca COVID-19 vaccine misinformation and fear mongering on Twitter. Public Health 2021 Nov;200:4-6 [doi: 10.1016/j.puhe.2021.08.019] [Medline: 34628307]

80. Calac AJ, Haupt MR, Li Z, Mackey T. Spread of COVID-19 Vaccine Misinformation in the Ninth Inning: Retrospective Observational Infodemic Study. JMIR Infodemiology 2022;2:e33587 [doi: 10.2196/33587] [Medline: 35320982]

81. Jiang LC, Chu TH, Sun M. Characterization of Vaccine Tweets During the Early Stage of the COVID-19 Outbreak in the United States: Topic Modeling Analysis. JMIR Infodemiology 2021;1:e25636 [doi: 10.2196/25636] [Medline: 34604707]

82. Ruiz JB, Bell RA. Predictors of intention to vaccinate against COVID-19: Results of a nationwide survey. Vaccine 2021 Feb 12;39:1080-1086 [doi: 10.1016/j.vaccine.2021.01.010] [Medline: 33461833]

83. An L, Russell DM, Mihalcea R, Bacon E, Huffman S, Resnicow K. Online Search Behavior Related to COVID-19 Vaccines: Infodemiology Study. JMIR Infodemiology 2021;1:e32127 [doi: 10.2196/32127] [Medline: 34841200]

84. Chen Y, Chen Y, Yang K, Lai F, Huang C, Chen Y, et al. The Prevalence and Impact of Fake News on COVID-19 Vaccination in Taiwan: Retrospective Study of Digital Media. J Med Internet Res 2022 Apr 26;24:e36830 [doi: 10.2196/36830] [Medline: 35380546]

85. Islam MS, Kamal AM, Kabir A, Southern DL, Khan SH, Hasan SMM, et al. COVID-19 vaccine rumors and conspiracy theories: The need for cognitive inoculation against misinformation to improve vaccine adherence. PLoS One 2021;16:e0251605 [doi: 10.1371/journal.pone.0251605] [Medline: 33979412]

86. Savoia E, Harriman NW, Piltch-Loeb R, Bonetti M, Toffolutti V, Testa MA. Exploring the Association between Misinformation Endorsement, Opinions on the Government Response, Risk Perception, and COVID-19 Vaccine Hesitancy in the US, Canada, and Italy. Vaccines (Basel) 2022 Apr 23;10:671 [doi: 10.3390/vaccines10050671] [Medline: 35632427]

88. Hammad AM, Al-Qerem W, Abu Zaid A, Khdair SI, Hall FS. Misconceptions Related to COVID 19 Vaccines Among the Jordanian Population: Myth and Public Health. Disaster Med Public Health Prep 2022 Jun 08:1-8 [doi: 10.1017/dmp.2022.143] [Medline: 35673791]

89. Eshel Y, Kimhi S, Marciano H, Adini B. Conspiracy claims and secret intentions as predictors of psychological coping and vaccine uptake during the COVID-19 pandemic. J Psychiatr Res 2022 Jul;151:311-318 [doi: 10.1016/j.jpsychires.2022.04.042] [Medline: 35526447]

90. Hornsey MJ, Chapman CM, Alvarez B, Bentley S, Salvador Casara BG, Crimston CR, et al. To what extent are conspiracy theorists concerned for self versus others? A COVID-19 test case. Eur J Soc Psychol 2021 Mar;51:285-293 [doi: 10.1002/ejsp.2737] [Medline: 33821057]

91. Loomba S, de Figueiredo A, Piatek SJ, de Graaf K, Larson HJ. Measuring the impact of COVID-19 vaccine misinformation on vaccination intent in the UK and USA. Nat Hum Behav 2021 Mar;5:337-348. [doi: 10.1038/s41562-021-01056-1] [Medline: 33547453]

92. Sallam M, Dababseh D, Eid H, Al-Mahzoum K, Al-Haidar A, Taim D, et al. High Rates of COVID-19 Vaccine Hesitancy and Its Association with Conspiracy Beliefs: A Study in Jordan and Kuwait among Other Arab Countries. Vaccines (Basel) 2021 Jan 12;9:42 [doi: 10.3390/vaccines9010042] [Medline: 33445581]

93. Andrade G. Covid-19 vaccine hesitancy, conspiracist beliefs, paranoid ideation and perceived ethnic discrimination in a sample of University students in Venezuela. Vaccine 2021 Nov 16;39:6837-6842 [doi: 10.1016/j.vaccine.2021.10.037] [Medline: 34711439]

94. Manby L, Dowrick A, Karia A, Maio L, Buck C, Singleton G, et al. Healthcare workers’ perceptions and attitudes towards the UK’s COVID-19 vaccination programme: a rapid qualitative appraisal. BMJ Open 2022 Feb 15;12:e051775. [doi: 10.1136/bmjopen-2021-051775]

95. Mahmud MR, Bin Reza R, Ahmed SZ. The effects of misinformation on COVID-19 vaccine hesitancy in Bangladesh. GKMC 2021 Oct 24:ahead-of-print. [doi: 10.1108/gkmc-05-2021-0080]

96. Buturoiu R, Udrea G, Oprea D, Corbu N. Who Believes in Conspiracy Theories about the COVID-19 Pandemic in Romania? An Analysis of Conspiracy Theories Believers’ Profiles. Societies 2021 Nov 13;11:138. [doi: 10.3390/soc11040138]

97. Jensen EA, Pfleger A, Herbig L, Wagoner B, Lorenz L, Watzlawik M. What Drives Belief in Vaccination Conspiracy Theories in Germany? Front. Commun 2021 May 25;6:105. [doi: 10.3389/fcomm.2021.678335]

98. Caycho-Rodríguez T, Ventura-León J, Valencia PD, Vilca LW, Carbajal-León C, Reyes-Bossio M, et al. What Is the Support for Conspiracy Beliefs About COVID-19 Vaccines in Latin America? A Prospective Exploratory Study in 13 Countries. Front. Psychol 2022 May 6;13:105. [doi: 10.3389/fpsyg.2022.855713]

99. Exline JJ, Pait KC, Wilt JA, Schutt WA. Demonic and Divine Attributions around COVID-19 Vaccines: Links with Vaccine Attitudes and Behaviors, QAnon and Conspiracy Beliefs, Anger, Spiritual Struggles, Religious and Political Variables, and Supernatural and Apocalyptic Beliefs. Religions 2022 Jun 06;13:519. [doi: 10.3390/rel13060519]

100. Lin F, Chen X, Cheng EW. Contextualized impacts of an infodemic on vaccine hesitancy: The moderating role of socioeconomic and cultural factors. Information Processing & Management 2022 Sep;59:103013. [doi: 10.1016/j.ipm.2022.103013]

101. Asadi Faezi N, Gholizadeh P, Sanogo M, Oumarou A, Mohamed MN, Cissoko Y, et al. Peoples' attitude toward COVID-19 vaccine, acceptance, and social trust among African and Middle East countries. Health Promot Perspect 2021;11:171-178 [doi: 10.34172/hpp.2021.21] [Medline: 34195040]

102. Aloweidi A, Bsisu I, Suleiman A, Abu-Halaweh S, Almustafa M, Aqel M, et al. Hesitancy towards COVID-19 Vaccines: An Analytical Cross-Sectional Study. Int J Environ Res Public Health 2021 May 12;18:5111 [doi: 10.3390/ijerph18105111] [Medline: 34065888]

103. Pierri F, Perry B, DeVerna M, Yang K, Flammini A, Menczer F, et al. Online misinformation is linked to early COVID-19 vaccination hesitancy and refusal. Sci Rep 2022 Apr 26;12:5966 [doi: 10.1038/s41598-022-10070-w] [Medline: 35474313]

104. Sharevski F, Alsaadi R, Jachim P, Pieroni E. Misinformation warnings: Twitter's soft moderation effects on COVID-19 vaccine belief echoes. Comput Secur 2022 Mar;114:102577 [doi: 10.1016/j.cose.2021.102577] [Medline:34934255]

105. Marchlewska M, Hamer K, Baran M, Górska P, Kaniasty K. COVID-19: Why Do People Refuse Vaccination? The Role of Social Identities and Conspiracy Beliefs: Evidence from Nationwide Samples of Polish Adults. Vaccines (Basel) 2022 Feb 10;10:268 [doi: 10.3390/vaccines10020268] [Medline: 35214726]

106. Perlis RH, Ognyanova K, Santillana M, Lin J, Druckman J, Lazer D, et al. Association of Major Depressive Symptoms With Endorsement of COVID-19 Vaccine Misinformation Among US Adults. JAMA Netw Open 2022 Jan 04;5:e2145697 [doi: 10.1001/jamanetworkopen.2021.45697] [Medline: 35061036]

107. Romer D, Jamieson KH. Conspiracy theories as barriers to controlling the spread of COVID-19 in the U.S. Soc Sci Med 2020 Oct;263:113356 [doi: 10.1016/j.socscimed.2020.113356] [Medline: 32967786]

108. Jennings W, Stoker G, Bunting H, Valgaresson VO, Gaskell J, Devine D, et al. Lack of Trust, Conspiracy Beliefs, and Social Media Use Predict COVID-19 Vaccine Hesitancy. Vaccines (Basel) 2021 Jun 03;9:593 [doi: 10.3390/vaccines9060593] [Medline: 34204971]

109. Kumar M, Madhumathi J, Gayathri K, A Rozario AG, Vijayaprabha R, Balusamy M, et al. Community voices around COVID-19 vaccine in Chennai, India: A qualitative exploration during early phase of vaccine rollout. Indian J Med Res 2022;155:451-460. [doi: 10.4103/ijmr.ijmr_668_22] [Medline: 35975352]

110. Baines A, Ittefaq M, Abwao M. #Scamdemic, #Plandemic, or #Scaredemic: What Parler Social Media Platform Tells Us about COVID-19 Vaccine. Vaccines (Basel) 2021 Apr 22;9:421 [doi: 10.3390/vaccines9050421] [Medline:33922343]

111. Saini V, Liang L, Yang Y, Le HM, Wu C. The Association Between Dissemination and Characteristics of Pro-/Anti-COVID-19 Vaccine Messages on Twitter: Application of the Elaboration Likelihood Model. JMIR Infodemiology 2022;2:e37077 [doi: 10.2196/37077] [Medline: 35783451]

112. Lanyi K, Green R, Craig D, Marshall C. COVID-19 Vaccine Hesitancy: Analysing Twitter to Identify Barriers to Vaccination in a Low Uptake Region of the UK. Front Digit Health 2021;3:804855 [doi: 10.3389/fdgth.2021.804855] [Medline: 35141699]

113. Larrondo-Ureta A, Fernández S, Morales-i-Gras J. Desinformación, vacunas y Covid-19. Análisis de la infodemia y la conversación digital en Twitter. RLCS 2021 Jun 07:1-18. [doi: 10.4185/rlcs-2021-1504]

114. Kalichman S, Eaton L, Earnshaw V, Brousseau N. Faster than warp speed: early attention to COVD-19 by anti-vaccine groups on Facebook. J Public Health (Oxf) 2022 Mar 07;44:e96-e105 [doi: 10.1093/pubmed/fdab093] [Medline: 33837428]

115. Wawrzuta D, Klejdysz J, Jaworski M, Gotlib J, Panczyk M. Attitudes toward COVID-19 Vaccination on Social Media: A Cross-Platform Analysis. Vaccines (Basel) 2022 Jul 27;10:1190 [doi: 10.3390/vaccines10081190] [Medline: 35893839]

116. Jin Q, Raza SH, Yousaf M, Zaman U, Siang JMLD. Can Communication Strategies Combat COVID-19 Vaccine Hesitancy with Trade-Off between Public Service Messages and Public Skepticism? Experimental Evidence from Pakistan. Vaccines (Basel) 2021 Jul 07;9:757 [doi: 10.3390/vaccines9070757] [Medline: 34358173]

117. Roozenbeek J, Schneider CR, Dryhurst S, Kerr J, Freeman ALJ, Recchia G, et al. Susceptibility to misinformation about COVID-19 around the world. R Soc Open Sci 2020 Oct;7:201199 [doi: 10.1098/rsos.201199] [Medline: 33204475]

118. Helfers A, Ebersbach M. The differential effects of a governmental debunking campaign concerning COVID-19 vaccination misinformation. Journal of Communication in Healthcare 2022 Mar 17:1-9. [doi: 10.1080/17538068.2022.2047497]

119. Vivion M, Anassour Laouan Sidi E, Betsch C, Dionne M, Dubé E, Driedger SM, et al. Prebunking messaging to inoculate against COVID-19 vaccine misinformation: an effective strategy for public health. Journal of Communication in Healthcare 2022 Mar 04;15:232-242. [doi: 10.1080/17538068.2022.2044606]

120. Basch CH, Hillyer GC, Zagnit EA, Basch CE. YouTube coverage of COVID-19 vaccine development: implications for awareness and uptake. Hum Vaccin Immunother 2020 Nov 01;16:2582-2585 [doi: 10.1080/21645515.2020.1790280] [Medline: 32701403]

121. Chan C, Sounderajah V, Daniels E, Acharya A, Clarke J, Yalamanchili S, et al. The Reliability and Quality of YouTube Videos as a Source of Public Health Information Regarding COVID-19 Vaccination: Cross-sectional Study. JMIR Public Health Surveill 2021 Jul 08;7:e29942 [doi: 10.2196/29942] [Medline: 34081599]

122. Criss S, Nguyen TT, Norton S, Virani I, Titherington E, Tillmanns EL, et al. Advocacy, Hesitancy, and Equity: Exploring U.S. Race-Related Discussions of the COVID-19 Vaccine on Twitter. Int J Environ Res Public Health 2021 May 26;18:5693 [doi: 10.3390/ijerph18115693] [Medline: 34073291]

123. Ginossar T, Cruickshank IJ, Zheleva E, Sulskis J, Berger-Wolf T. Cross-platform spread: vaccine-related content, sources, and conspiracy theories in YouTube videos shared in early Twitter COVID-19 conversations. Hum Vaccin Immunother 2022 Dec 31;18:1-13 [doi: 10.1080/21645515.2021.2003647] [Medline: 35061560]

124. Yousuf H, van der Linden S, Bredius L, Ted van Essen GA, Sweep G, Preminger Z, et al. A media intervention applying debunking versus non-debunking content to combat vaccine misinformation in elderly in the Netherlands: A digital randomised trial. EClinicalMedicine 2021 May;35:100881 [doi: 10.1016/j.eclinm.2021.100881] [Medline: 34124631]

125. Lu S, Zhong L. From Believing to Sharingxamining the Effects of Partisan Media's Correction of COVID-19 Vaccine Misinformation. INTERNATIONAL JOURNAL OF COMMUNICATION. 2022 2022 2022 Jun 15:16.

126. Talabi F, Ugbor I, Talabi M, Ugwuoke J, Oloyede D, Aiyesimoju A, et al. Effect of a social media-based counselling intervention in countering fake news on COVID-19 vaccine in Nigeria. Health Promot Int 2022 Apr 29;37:daab140. [doi: 10.1093/heapro/daab140] [Medline: 34510187]

127. Piltch-Loeb R, Su M, Hughes B, Testa M, Goldberg B, Braddock K, et al. Testing the Efficacy of Attitudinal Inoculation Videos to Enhance COVID-19 Vaccine Acceptance: Quasi-Experimental Intervention Trial. JMIR Public Health Surveill 2022 Jun 20;8:e34615 [doi: 10.2196/34615] [Medline: 35483050]

128. Dai Y, Jia W, Fu L, Sun M, Jiang LC. The effects of self-generated and other-generated eWOM in inoculating against misinformation. Telematics and Informatics 2022 Jul;71:101835. [doi: 10.1016/j.tele.2022.101835]
