# Supplementary material for: The Prevalence, Features, Influencing Factors, and Solutions for COVID-19 Vaccine Misinformation: Systematic Review
Source: JMIR Public Health Surveill. 2023 Jan 11;9:e40201. doi: 10.2196/40201 (PMC9838721; doi:10.2196/40201)
Supplement: Multimedia Appendix 5 [file publichealth_v9i1e40201_app5.docx]

## Appendix5. Prevalence of COVID-19 Vaccine-related Misinformation by Study Design

| Study design | Region | Study Phase | Data source | Contents | Type of misinformation | Prevalence of Misinformation | Reference |
| --- | --- | --- | --- | --- | --- | --- | --- |
| **Surveys on general population** | African | Phase 2 | Respondents in Africa | No vaccine No enough evidence Alter DNA Infertility and offspring Immune from infection Not risk group | Conspiracy; Concerns on vaccine safety and efficacy; No need for vaccine | Conspiracy: 13.0%; Concerns on vaccine safety and efficacy: 23.8%; No need for vaccine: 16.0% | Lamptey et al., 2022 [50] |
|  | African | Phase 2 | Adults in Ethiopia, Addis Ababa, Akaki Kality sub-city | Mild COVID-19 Cause COVID-19 Bioweapon | Conspiracy; Concerns on vaccine safety and efficacy; No need for vaccine | Conspiracy: 41.0%;  Concerns on vaccine safety and efficacy: 11.5%;  No need for vaccine: 3.8% | Dereje et al., 2021 [37] |
|  | American | Phase 1 | USA adults | Financial incentive in vaccine developing | Conspiracy | Conspiracy: 14.8% | Romer and Jamieson, 2020 [107] |
|  | American | Phase 1 | USA adults | Financial incentive in vaccine developing | Conspiracy | Conspiracy: 17.4% | Romer and Jamieson, 2021 [1] |
|  | American | Phase 2 | Adults in 13 Latin American countries | Fabricated vaccine efficacy Evidence of unsafety is covered up | Conspiracy | Conspiracy: 24~45% (Chile: 24%; Cuba: 25%; Peru: 40%; Guatemala: 45%) | Caycho-Rodríguez et al.,2022 [98] |
|  | American | Phase 2 | Florida adults | Device for track/control Depopulation COVID-19 for vaccine Live virus Alter DNA Infertility and offspring Cause death Against government control | Conspiracy; Concerns on vaccine safety and efficacy; Liberty | Conspiracy: 18.8~42.8%; Concerns on vaccine safety and efficacy: 6.0%; Liberty: 36.3% | Neely et al., 2021 [56] |
|  | American, European | Phase 1 | Participants from five countries | COVID-19 for vaccine | Conspiracy | Conspiracy: 12.95%~22.9% (Ireland: 18%;USA: 18.14%; Mexico: 22.29%; Spain: 18.86%; UK-April: 12.95%; UK-May: 16.17%) | Roozenbeek et al., 2020 [117] |
|  | American, European, Western Pacific | Phase 1 | Participants from eight nations | Device for track/control | Conspiracy | Conspiracy: 14.3% | Hornsey et al., 2021 [90] |
|  | American, European, Western Pacific | Phase 2 | Representative respondents from 6 countries | Population control and new world order Alter DNA Financial incentive in vaccine developing | Conspiracy; Concerns on vaccine safety and efficacy | Conspiracy: 34.0~35.9% | Lin et al., 2022 [100] |
|  | Eastern Mediterranean | Phase 1 | University students in Jordan | COVID-19 for vaccine Device for track/control Infertility and offspring | Conspiracy; Concerns on vaccine safety and efficacy | Conspiracy: 11~29.7%; Concerns on vaccine safety and efficacy: 45.4% | Sallam et al., 2021 [60] |
|  | Eastern Mediterranean | Phase 1 | Saudi Arabia residents | No vaccine Not risk group | Conspiracy; No need for vaccine | Conspiracy: 18%;  No need for vaccine: 6.5% | Magadmi and Kamel, 2021 [29] |
|  | Eastern Mediterranean | Phase 2 | Pakistan respondents >15 years | Cause COVID-19 Worse than COVID-19 Alter DNA Infertility and offspring Cause other diseases Unnecessary | Concerns on vaccine safety and efficacy; No need for vaccine | Concerns on vaccine safety and efficacy:11~55%; No need for vaccine: 28% | Magadmi and Kamel, 2021 [29] |
|  | Eastern Mediterranean | Phase 2 | Jodan respondents | Device for track/control Political incentive in vaccine developing Rush in development Depopulation Vaccine would not work Cause COVID-19 Worse than COVID-19 Alter DNA Infertility and offspring Cause other diseases Alternative Not risk group | Conspiracy; Concerns on vaccine safety and efficacy; No need for vaccine; Morality | Conspiracy: 12.7~54% Concerns on vaccine safety and efficacy: 14~55.4%; No need for vaccine: 24.1~28.1%; Morality: 20.6% | Abbas et al., 2022 [70] |
|  | Eastern Mediterranean | Phase 2 | Medical personnel | Alter DNA Infertility and offspring Cause other diseases Poison | Conspiracy; Concerns on vaccine safety and efficacy | Conspiracy: 5.2~12.9%;  Concerns on vaccine safety and efficacy: 5.2~8.4% | Hammad et al., 2022 [88] |
|  |  |  | Non-medical adults | Alter DNA Infertility and offspring Cause other diseases Poison | Conspiracy; Concerns on vaccine safety and efficacy | Conspiracy: 7.8%~17.8%;  Concerns on vaccine safety and efficacy: 8.9%~10.6% | Aloweidi et al., 2021 [102] |
|  | Eastern Mediterranean | Phase 2 | Pakistan residents | Cause death Infertility and offspring Device for track/control Depopulation Conspiracy of a certain country/region (e.g., African region, Muslim nations, etc.) Rush in development | Conspiracy | Conspiracy: 9.3%~28.4% | Arshad et al., 2021 [74] |
|  | Eastern Mediterranean | Phase 2 | Residents of Jordan, Kuwait and Saudi Arabia | Device for track/control COVID-19 for vaccine Infertility and offspring | Conspiracy; Concerns on vaccine safety and efficacy | Conspiracy: 16.5%~48.4% (Jordan: 23.2~38.3%; Kuwait: 27.5%~48.4%; Saudi Arbia:16.5%~30.7%) | Sallam et al., 2021 [92] |
|  | Eastern Mediterranean | Phase 2 | Students of the American University of Beirut | Population control and new world order Fabricated vaccine efficacy Cover up side effect data Unnecessary Worse than COVID-19 Freedom | Conspiracy; Concerns on vaccine safety and efficacy; No need for vaccine; Liberty | Conspiracy: 8%~10% Concerns on vaccine safety and efficacy: 5% no need for vaccine: 14% Liberty: 6% | Hamdan et al., 2021 [42] |
|  | European | Phase 1 | Germany residents | COVID-19 for vaccine | Conspiracy | Conspiracy: 15% | Jensen et al., 2021 [97] |
|  | European | Phase 2 | Hungary adults | Device for track/control Population control and new world order COVID-9 for vaccine | Conspiracy | Conspiracy: 5~14% | Bíró‑Nagy and Szász, 2022 [32] |
|  | South-East Asian | NA | Indian adults | Rush in development Ineffective in certain group Cause other diseases | Conspiracy;  Concerns on vaccine safety and efficacy | Conspiracy: 2.78%;  Concerns on vaccine safety and efficacy: 2.78~6.48% | Bíró‑Nagy and Szász, 2022 [32] |
|  | South-East Asian | Phase 2 | Bangladeshi adults | Device for track/control Population control and new world order No enough evidence Alter DNA Infertility and offspring Cause other diseases Religion | Conspiracy; Concerns on vaccine safety and efficacy; Morality | Conspiracy: 2.5~40.5%; Concerns on vaccine safety and efficacy: 11.1~25.3%; Morality: 1.4% | Kulkarni et al., 2021 [47] |
|  | Western Pacific | Phase 2 | Chinese adults | Fabricated vaccine efficacy Cover up side effect data | Conspiracy | Conspiracy: 14.2~19.5% | Mahmud et al., 2021 [95] |
|  | World-wide | Phase 2 | Participants from 42 nations | Alter DNA | Concerns on vaccine safety and efficacy | Concerns on vaccine safety and efficacy: 27.61% | Yang et al., 2021 [68] |
| **Surveys on antivaccine/vaccine hesitant group** | American | Phase 1 | USA English-speaking adults | Vaccine would not work Alternative Immune from infection Not risk group | Concerns on vaccine safety and efficacy; No need for vaccine | Concerns on vaccine safety and efficacy: 20.1%;  No need for vaccine: 6.3%~17.8% | Faezi et al., 2021 [101] |
|  | American | Phase 2 | USA adults who think vaccine not going to be safe | Device for track/control Population control and new world order Unnecessary Poison Alter DNA | Conspiracy; Concerns on vaccine safety and efficacy; No need for vaccine | Conspiracy: 11.1~18.6%; Concerns on vaccine safety and efficacy: 12.2%; No need for vaccine:14.1% | Ruiz and Bell, 2021 [82] |
|  | Eastern Mediterranean | Phase 1 | Saudi Arabia residents | No vaccine Not risk group | Conspiracy; No need for vaccine | Conspiracy: 22.2%;  No need for vaccine: 10.3% | Kricorian et al., 2022 [31] |
|  | European | Phase 1 | Dutch vaccine hesitant people | Poison Immune from infection | Concerns on vaccine safety and efficacy; No need for vaccine | Concerns on vaccine safety and efficacy: 96.7%; No need for vaccine: 70.4% | Yousuf et al., 2021 [30] |
|  | European | Phase 2 | Hungary adults | Device for track/control Population control and new world order COVID-10 for vaccine | Conspiracy | Conspiracy: 10~21% | Bíró‑Nagy and Szász, 2022 [32] |
|  | World-wide | Phase 1 & 2 | Participants from 13 countries | Cause COVID-19 Not risk group | Conspiracy; Concerns on vaccine safety and efficacy; No need for vaccine | Conspiracy: 6.0%~11.6% ( All LMICs: 11.6%; Russia:21.4%; USA 6.0%);  Concerns on vaccine safety and efficacy: 13.9%~17.6% (All LMICs: 17.6%; Russia:13.9%);  No need for vaccine: 6.4%~8.7% (ll LMICs: 8.7%; Russia:6.4%) | Arce et al., 2021 [76] |
| **Internet-based studies on general online data** | African | Phase 1 & 2 | Social media: Twitter | Freedom Financial incentive in vaccine developing | Conspiracy; Concerns on vaccine safety and efficacy; Liberty | Conspiracy: 5.3%; Concerns on vaccine safety and efficacy: 0.4~7.7%; Liberty: 41.1% | Al-Rawi et al., 2022 [71] |
|  | American | Phase 1 | Social media: Twitter | Other conspiracy about authorities, pharmacy company, or certain people | Conspiracy | Conspiracy: 4.9% | Jiang et al.,2021 [81] |
|  | American | Phase 1 & 2 | Social media: Twitter | Device for track/control Conspiracy of ethnic minority (e.g., Asian, black people) Alternative Humor | Conspiracy; No need for vaccine; Humor | Misinformation in general: 15% | Criss et al., 2021 [122] |
|  | American | Phase 2 | Google trends | Device for track/control Alter DNA Infertility and offspring Cause COVID-19 | Conspiracy; Concerns on vaccine safety and efficacy | Misinformation in general: <1% | An et al., 2021 [83] |
|  | European | Phase 2 | Social media: Twitter | No enough evidence Other conspiracy about authorities, pharmacy company, or certain people COVID-19 denial Unnecessary Religion | Conspiracy; Concerns on vaccine safety and efficacy; No need for vaccine; Morality | Conspiracy: 5.3%~21.7%;  Concerns on vaccine safety and efficacy: 11.1%;  No need for vaccine: 10.1%;  Morality: 3.9% | Küçükali et al., 2021 [46] |
|  | Western Pacific | Phase 2 & 3 | Internet news media sources | NA | Misinformation in general | Misininformation in general: 33.7%~41.3% | Chen et al., 2022 [84] |
|  | World-wide | NA | Social media: TikTok | No vaccine Humor | Conspiracy; Humor | Conspiracy: 3%;  Humor: 26% | Basch et al., 2021 [34] |
|  | World-wide | Phase 1 | Social media: YouTube | NA | Concerns on vaccine safety and efficacy | Concerns on vaccine safety and efficacy: 4.2% | Chan et al., 2021 [121] |
|  | World-wide | Phase 1 | Social media: YouTube | COVID-19 denial | Conspiracy | Conspiracy: 2% | Basch et al., 2020 [120] |
|  | World-wide | Phase 1 | Social media: Reddit | NA | Conspiracy | Conspiracy: 12.44% | Wu et al., 2021 [67] |
|  | World-wide | Phase 1 | Social media: Reddit | NA | Conspiracy | Conspiracy: 40% | Kumar et al., 2022 [109] |
|  | World-wide | Phase 1 & 2 | Database of online article | NA | Misinformation in general | Misinformation in general: 0.1% | Lurie et al., 2022 [54] |
|  | World-wide | Phase 1 & 2 | Social media: Youtube | NA | Misinformation in general | Misinformation in general: 10% | Laforet et al., 2022 [49] |
|  | World-wide | Phase 1 & 2 | Social media: Youtube | Vaccine would not work Infertility and offspring Underline disease Immune from infection | Concerns on vaccine safety and efficacy; No need for vaccine | Misinformation in general: 10.7% | Li et al., 2022 [52] |
|  | World-wide | Phase 1 & 2 | Social media: YouTube | Other conspiracy about authorities, pharmacy company, or certain people Religion | Conspiracy; Morality | Misinformation in general: 1.7% | Hernández-García et al., 2021 [43] |
|  | World-wide | Phase 2 | Social media: Twitter | Other conspiracy about authorities, pharmacy company, or certain people Rejection in registration | Conspiracy | Conspiracy: 5.3% | Jemielniak and Krempovych, 2021 [79] |
| **Internet-based studies on antivaccine/vaccine hesitant data** | American | Phase 1 & 2 | Social media: Parler | Depopulation Ignore Consent | Conspiracy; Liberty | Conspiracy: 23%; liberty: 5% | Baines et al., 2021 [110] |
|  | American | Phase 2 | Social media: Twitter | Freedom Political incentive in vaccine developing Alter DNA Side-effect responsibility Mild COVID-19 | Conspiracy; Concerns on vaccine safety and efficacy; Liberty | Conspiracy: 32.4%;  Concerns on vaccine safety and efficacy: 3.1%~26.3% | Griffith et al., 2021 [41] |
|  | American | Phase 2 & 3 | Google image | Against government control Freedom | Liberty | Liberty: 46.0% | Liao et al., 2022 [53] |
|  | European | Phase 2 | Social media: Twitter | Financial incentive in vaccine developing Cause other diseases New strain for cover-up | Conspiracy; Concerns on vaccine safety and efficacy | Misinformation in general: 27.5% | Lanyi et al., 2021 [112] |
|  | European | Phase 2 | Social media: Facebook | Paid for promotion Human experiment Religion Against government control | Conspiracy; Concerns on vaccine safety and efficacy; Morality; Liberty | Conspiracy: 12.5%; Concerns on vaccine safety and efficacy: 14%; Morality: 2%; Liberty: 6% | Obreja et al., 2022 [57] |
|  | European | Phase 2 & 3 | Social media: Twitter | Financial incentive in vaccine developing Device for track/control Rush in development Playacting in promotion Vaccine would not work Worse than COVID-19 Side-effect responsibility Alternative Mild COVID-19 Freedom | Conspiracy; Concerns on vaccine safety and efficacy; No need for vaccine; Liberty | Conspiracy: 2.7~6.9%; Concerns on vaccine safety and efficacy: 1.3~18.6%; No need for vaccine: 0.5%; Liberty: 15.6% | Wawrzuta et al., 2022 [115] |
|  | World-wide | NA | Multiple social media: YouTube, Twitter, Facebook, and Instagram | Other conspiracy about authorities, pharmacy company, or certain people Unspecified danger Religion Freedom | Conspiracy; Concerns on vaccine safety and efficacy; Morality; Liberty; Morality | Conspiracy: 20.4%;  Concerns on vaccine safety and efficacy: 12.1%;  Liberty: 7.8%~9.3%;  Morality: 10.4% | Hughes et al., 2021 [73] |
|  | World-wide | Phase 1 | Multiple: Google, Google Fact Check, Facebook, YouTube, Twitter, fact-checking agency websites, and websites of television and newspaper | Device for track/control Conspiracy of a certain country/region (e.g., African region, Muslim nations, etc.) Depopulation Device for track/control  Rush in development Ineffective claim from authority  Infertility and offspring  Vaccine would not work Alter DNA  Poison  Cause death  Conspiracy of other certain groups (e.g., Muslims, elder people, less educated people, low-income group, etc.) Alternative Mild COVID-19 Refusal exaggeration Overstatement Fetal tissue Human experiment Mandatory vaccination | Conspiracy; Concerns on vaccine safety and efficacy; No need for vaccine; Morality; Liberty; Overstatement | Conspiracy: 9%;  Concerns on vaccine safety and efficacy: 9.3%;  No need for vaccine: 3.7% | Islam et al., 2021 [85] |
|  | World-wide | Phase 1 | Social media: Facebook | Financial incentive in vaccine developing Alter DNA | Conspiracy; Concerns on vaccine safety and efficacy | Misinformation in general: 17~34% | Kalichman et al, 2021 [114] |
|  | World-wide | Phase 1 | Social media: Twitter | No vaccine Alter DNA Poison | Conspiracy; Concerns on vaccine safety and efficacy | Conspiracy: 23.5%;  Concerns on vaccine safety and efficacy: 10.5% | Thelwall et al., 2021 [62] |
|  | World-wide | Phase 1 & 2 | Database of online article | Device for track/control Population control and new world order Paid for promotion Human experiment Fetal tissue Alter DNA Cause other diseases Poison | Conspiracy; Concerns on vaccine safety and efficacy; No need for vaccine; Morality | Conspiracy: 55.4%; Concerns on vaccine safety: 44.8% | Lurie et al., 2022 [54] |
|  | World-wide | Phase 1 & 2 | Social media: Twitter | Depopulation | Conspiracy | Conspiracy: 3.90%~5.04% | Muric et al., 2021 [55] |
|  | World-wide | Phase 2 | Social media: Twitter | Device for track/control Population control and new world order Depopulation Other conspiracy about authorities, pharmacy company, or certain people Alter DNA Infertility and offspring | Conspiracy; Concerns on vaccine safety and efficacy; Morality | Misinformation in general: 56% | Calac et al., 2022 [80] |
|  | World-wide | Phase 2 | Social media: Twitter | Device for track/control Population control and new world order Depopulation Vaccine would not work Alternative | Conspiracy; Concerns on vaccine safety and efficacy; No need for vaccine | Conspiracy: 16.97%;  Concerns on vaccine safety and efficacy: 19.67%;  No need for vaccine: 2.7% | Herrera-Peco et al., 2021 [15] |
|  | World-wide | Phase 2 | Social media: Twitter | NA | Misinformation in general | Misinformation in general: 9.4% | Charquero-Ballester et al., 2021 [36] |

## References:

1. Romer D, Jamieson KH. Patterns of Media Use, Strength of Belief in COVID-19 Conspiracy Theories, and the Prevention of COVID-19 From March to July 2020 in the United States: Survey Study. J Med Internet Res 2021 Apr 27;23:e25215 [doi: 10.2196/25215] [Medline: 33857008]

15. Herrera-Peco I, Jiménez-Gómez B, Romero Magdalena CS, Deudero JJ, García-Puente M, Benítez De Gracia E, et al. Antivaccine Movement and COVID-19 Negationism: A Content Analysis of Spanish-Written Messages on Twitter. Vaccines (Basel) 2021 Jun 15;9:656 [doi: 10.3390/vaccines9060656] [Medline: 34203946]

29. Magadmi RM, Kamel FO. Beliefs and barriers associated with COVID-19 vaccination among the general population in Saudi Arabia. BMC Public Health 2021 Jul 21;21:1438. [doi: 10.1186/s12889-021-11501-5] [Medline: 34289817]

30. Yousuf H, van der Linden S, van Essen T, Gommers D, Scherder E, Narula J, et al. Dutch Perspectives Toward Governmental Trust, Vaccination, Myths, and Knowledge About Vaccines and COVID-19. JAMA Netw Open 2021 Dec 01;4:e2140529 [doi: 10.1001/jamanetworkopen.2021.40529] [Medline: 34967887]

31. Kricorian K, Civen R, Equils O. COVID-19 vaccine hesitancy: misinformation and perceptions of vaccine safety. Hum Vaccin Immunother 2022 Dec 31;18:1950504 [doi: 10.1080/21645515.2021.1950504] [Medline: 34325612]

32. Bíró-Nagy A, Szászi áJ. The roots of COVID-19 vaccine hesitancy: evidence from Hungary. J Behav Med 2022 May 14:1-16 [doi: 10.1007/s10865-022-00314-5] [Medline: 35567729]

34. Basch CH, Meleo-Erwin Z, Fera J, Jaime C, Basch CE. A global pandemic in the time of viral memes: COVID-19 vaccine misinformation and disinformation on TikTok. Hum Vaccin Immunother 2021 Aug 03;17:2373-2377 [doi: 10.1080/21645515.2021.1894896] [Medline: 33764283]

36. Charquero-Ballester M, Walter J, Nissen I, Bechmann A. Different types of COVID-19 misinformation have different emotional valence on Twitter. Big Data & Society 2021 Sep 22;8:205395172110412. [doi: 10.1177/20539517211041279]

37. Dereje N, Tesfaye A, Tamene B, Alemeshet D, Abe H, Tesfa N, et al. COVID-19 vaccine hesitancy in Addis Ababa, Ethiopia: a mixed-method study. BMJ Open 2022 May 30;12:e052432 [doi: 10.1136/bmjopen-2021-052432] [Medline: 35636790]

41. Griffith J, Marani H, Monkman H. COVID-19 Vaccine Hesitancy in Canada: Content Analysis of Tweets Using the Theoretical Domains Framework. J Med Internet Res 2021 Apr 13;23:e26874 [doi: 10.2196/26874] [Medline: 33769946]

42. Bou Hamdan M, Singh S, Polavarapu M, Jordan T, Melhem N. COVID-19 vaccine hesitancy among university students in Lebanon. Epidemiol. Infect 2021 Nov 02;149:e242. [doi: 10.1017/s0950268821002314]

43. Hernández-García I, Gascón-Giménez I, Gascón-Giménez A, Giménez-Júlvez T. Information in Spanish on YouTube about Covid-19 vaccines. Hum Vaccin Immunother 2021 Nov 02;17:3916-3921 [doi: 10.1080/21645515.2021.1957416] [Medline: 34375570]

46. Küçükali H, Ataç Ö, Palteki AS, Tokaç AZ, Hayran O. Vaccine Hesitancy and Anti-Vaccination Attitudes during the Start of COVID-19 Vaccination Program: A Content Analysis on Twitter Data. Vaccines (Basel) 2022 Jan 21;10:161 [doi: 10.3390/vaccines10020161] [Medline: 35214620]

47. Kulkarni M, Khurana K. Acceptance and myths regarding covid vaccination among general population. Medical Science 2022;26:ms255e2245 [doi: 10.54905/disssi/v26i124/ms255e2245]

49. Laforet PE, Basch CH, Tang H. Understanding the content of COVID-19 vaccination and pregnancy videos on YouTube: An analysis of videos published at the start of the vaccine rollout. Hum Vaccin Immunother 2022 Nov 30;18:2066935 [doi: 10.1080/21645515.2022.2066935] [Medline: 35507867]

50. Lamptey E, Senkyire EK, Dorcas S, Benita DA, Boakye EO, Ikome T, et al. Exploring the myths surrounding the COVID-19 vaccines in Africa: the study to investigate their impacts on acceptance using online survey and social media. Clin Exp Vaccine Res 2022 May;11:193-208 [doi: 10.7774/cevr.2022.11.2.193] [Medline: 35799880]

52. Li HOY, Pastukhova E, Brandts-Longtin O, Tan MG, Kirchhof MG. YouTube as a source of misinformation on COVID-19 vaccination: a systematic analysis. BMJ Glob Health 2022 Mar;7:e008334 [doi: 10.1136/bmjgh-2021-008334] [Medline: 35264318]

53. Liao TF. Understanding Anti-COVID-19 Vaccination Protest Slogans in the US. Front. Commun 2022 Jun 30;7:941872 [doi: 10.3389/fcomm.2022.941872]

54. Lurie P, Adams J, Lynas M, Stockert K, Carlyle RC, Pisani A, et al. COVID-19 vaccine misinformation in English-language news media: retrospective cohort study. BMJ Open 2022 Jun 01;12:e058956 [doi: 10.1136/bmjopen-2021-058956] [Medline: 35649595]

55. Muric G, Wu Y, Ferrara E. COVID-19 Vaccine Hesitancy on Social Media: Building a Public Twitter Data Set of Antivaccine Content, Vaccine Misinformation, and Conspiracies. JMIR Public Health Surveill 2021 Nov 17;7:e30642 [doi: 10.2196/30642] [Medline: 34653016]

56. Neely SR, Eldredge C, Ersing R, Remington C. Vaccine Hesitancy and Exposure to Misinformation: a Survey Analysis. J Gen Intern Med 2022 Jan;37:179-187 [doi: 10.1007/s11606-021-07171-z] [Medline: 34671900]

57. Obreja DM. Narrative communication regarding the Covid-19 vaccine: a thematic analysis of comments on Romanian official Facebook page "RO Vaccinare". SN Soc Sci 2022;2:119 [doi: 10.1007/s43545-022-00427-3] [Medline: 35875608]

60. Sallam M, Dababseh D, Eid H, Hasan H, Taim D, Al-Mahzoum K, et al. Low COVID-19 Vaccine Acceptance Is Correlated with Conspiracy Beliefs among University Students in Jordan. Int J Environ Res Public Health 2021 Mar 01;18:2407 [doi: 10.3390/ijerph18052407] [Medline: 33804558]

62. Thelwall M, Kousha K, Thelwall S. Covid-19 vaccine hesitancy on English-language Twitter. El Profesional de la Información 2021;30:1-13. [doi: 10.3145/epi.2021.mar.12]

67. Wu W, Lyu H, Luo J. Characterizing Discourse about COVID-19 Vaccines: A Reddit Version of the Pandemic Story. Health Data Sci 2021;2021:9837856 [doi: 10.34133/2021/9837856] [Medline: 36405359]

68. Yang Z, Luo X, Jia H. Is It All a Conspiracy? Conspiracy Theories and People's Attitude to COVID-19 Vaccination. Vaccines (Basel) 2021 Sep 22;9:1051 [doi: 10.3390/vaccines9101051] [Medline: 34696159]

70. Abbas SW, Zareen SF, Nisar S, Farooq A, Rasheed A, Saleem MU. COVID-19 Vaccines: Community Myths Vs Facts. Pakistan Armed Forces Medical Journal. 2022;72(2):497-500 [doi: 10.51253/pafmj.v72i2.6970] [Medline: 157247889]

71. Al-Rawi A, Fakida A, Grounds K. Investigation of COVID-19 Misinformation in Arabic on Twitter: Content Analysis. JMIR Infodemiology. 2022 Jul-Dec;2(2):e37007 [doi: 10.2196/37007] [Medline: 35915823]

73. Hughes B, Miller-Idriss C, Piltch-Loeb R, Goldberg B, White K, Criezis M, et al. Development of a Codebook of Online Anti-Vaccination Rhetoric to Manage COVID-19 Vaccine Misinformation. Int J Environ Res Public Health 2021 Jul 15;18:7556 [doi: 10.3390/ijerph18147556] [Medline: 34300005]

74. Arshad MS, Hussain I, Mahmood T, Hayat K, Majeed A, Imran I, et al. A National Survey to Assess the COVID-19 Vaccine-Related Conspiracy Beliefs, Acceptability, Preference, and Willingness to Pay among the General Population of Pakistan. Vaccines (Basel) 2021 Jul 01;9:720 [doi: 10.3390/vaccines9070720] [Medline: 34358136]

76. Solís Arce JS, Warren SS, Meriggi NF, Scacco A, McMurry N, Voors M, et al. COVID-19 vaccine acceptance and hesitancy in low- and middle-income countries. Nat Med 2021 Aug;27:1385-1394 [doi: 10.1038/s41591-021-01454-y] [Medline: 34272499]

79. Jemielniak D, Krempovych Y. An analysis of AstraZeneca COVID-19 vaccine misinformation and fear mongering on Twitter. Public Health 2021 Nov;200:4-6 [doi: 10.1016/j.puhe.2021.08.019] [Medline: 34628307]

80. Calac AJ, Haupt MR, Li Z, Mackey T. Spread of COVID-19 Vaccine Misinformation in the Ninth Inning: Retrospective Observational Infodemic Study. JMIR Infodemiology 2022;2:e33587 [doi: 10.2196/33587] [Medline: 35320982]

81. Jiang LC, Chu TH, Sun M. Characterization of Vaccine Tweets During the Early Stage of the COVID-19 Outbreak in the United States: Topic Modeling Analysis. JMIR Infodemiology 2021;1:e25636 [doi: 10.2196/25636] [Medline: 34604707]

82. Ruiz JB, Bell RA. Predictors of intention to vaccinate against COVID-19: Results of a nationwide survey. Vaccine 2021 Feb 12;39:1080-1086 [doi: 10.1016/j.vaccine.2021.01.010] [Medline: 33461833]

83. An L, Russell DM, Mihalcea R, Bacon E, Huffman S, Resnicow K. Online Search Behavior Related to COVID-19 Vaccines: Infodemiology Study. JMIR Infodemiology 2021;1:e32127 [doi: 10.2196/32127] [Medline: 34841200]

84. Chen Y, Chen Y, Yang K, Lai F, Huang C, Chen Y, et al. The Prevalence and Impact of Fake News on COVID-19 Vaccination in Taiwan: Retrospective Study of Digital Media. J Med Internet Res 2022 Apr 26;24:e36830 [doi: 10.2196/36830] [Medline: 35380546]

85. Islam MS, Kamal AM, Kabir A, Southern DL, Khan SH, Hasan SMM, et al. COVID-19 vaccine rumors and conspiracy theories: The need for cognitive inoculation against misinformation to improve vaccine adherence. PLoS One 2021;16:e0251605 [doi: 10.1371/journal.pone.0251605] [Medline: 33979412]

88. Hammad AM, Al-Qerem W, Abu Zaid A, Khdair SI, Hall FS. Misconceptions Related to COVID 19 Vaccines Among the Jordanian Population: Myth and Public Health. Disaster Med Public Health Prep 2022 Jun 08:1-8 [doi: 10.1017/dmp.2022.143] [Medline: 35673791]

90. Hornsey MJ, Chapman CM, Alvarez B, Bentley S, Salvador Casara BG, Crimston CR, et al. To what extent are conspiracy theorists concerned for self versus others? A COVID-19 test case. Eur J Soc Psychol 2021 Mar;51:285-293 [doi: 10.1002/ejsp.2737] [Medline: 33821057]

92. Sallam M, Dababseh D, Eid H, Al-Mahzoum K, Al-Haidar A, Taim D, et al. High Rates of COVID-19 Vaccine Hesitancy and Its Association with Conspiracy Beliefs: A Study in Jordan and Kuwait among Other Arab Countries. Vaccines (Basel) 2021 Jan 12;9:42 [doi: 10.3390/vaccines9010042] [Medline: 33445581]

95. Mahmud MR, Bin Reza R, Ahmed SZ. The effects of misinformation on COVID-19 vaccine hesitancy in Bangladesh. GKMC 2021 Oct 24:ahead-of-print. [doi: 10.1108/gkmc-05-2021-0080]

97. Jensen EA, Pfleger A, Herbig L, Wagoner B, Lorenz L, Watzlawik M. What Drives Belief in Vaccination Conspiracy Theories in Germany? Front. Commun 2021 May 25;6:105. [doi: 10.3389/fcomm.2021.678335]

98. Caycho-Rodríguez T, Ventura-León J, Valencia PD, Vilca LW, Carbajal-León C, Reyes-Bossio M, et al. What Is the Support for Conspiracy Beliefs About COVID-19 Vaccines in Latin America? A Prospective Exploratory Study in 13 Countries. Front. Psychol 2022 May 6;13:105. [doi: 10.3389/fpsyg.2022.855713]

100. Lin F, Chen X, Cheng EW. Contextualized impacts of an infodemic on vaccine hesitancy: The moderating role of socioeconomic and cultural factors. Information Processing & Management 2022 Sep;59:103013. [doi: 10.1016/j.ipm.2022.103013]

101. Asadi Faezi N, Gholizadeh P, Sanogo M, Oumarou A, Mohamed MN, Cissoko Y, et al. Peoples' attitude toward COVID-19 vaccine, acceptance, and social trust among African and Middle East countries. Health Promot Perspect 2021;11:171-178 [doi: 10.34172/hpp.2021.21] [Medline: 34195040]

102. Aloweidi A, Bsisu I, Suleiman A, Abu-Halaweh S, Almustafa M, Aqel M, et al. Hesitancy towards COVID-19 Vaccines: An Analytical Cross-Sectional Study. Int J Environ Res Public Health 2021 May 12;18:5111 [doi: 10.3390/ijerph18105111] [Medline: 34065888]

107. Romer D, Jamieson KH. Conspiracy theories as barriers to controlling the spread of COVID-19 in the U.S. Soc Sci Med 2020 Oct;263:113356 [doi: 10.1016/j.socscimed.2020.113356] [Medline: 32967786]

109. Kumar M, Madhumathi J, Gayathri K, A Rozario AG, Vijayaprabha R, Balusamy M, et al. Community voices around COVID-19 vaccine in Chennai, India: A qualitative exploration during early phase of vaccine rollout. Indian J Med Res 2022;155:451-460. [doi: 10.4103/ijmr.ijmr_668_22] [Medline: 35975352]

110. Baines A, Ittefaq M, Abwao M. #Scamdemic, #Plandemic, or #Scaredemic: What Parler Social Media Platform Tells Us about COVID-19 Vaccine. Vaccines (Basel) 2021 Apr 22;9:421 [doi: 10.3390/vaccines9050421] [Medline:33922343]

112. Lanyi K, Green R, Craig D, Marshall C. COVID-19 Vaccine Hesitancy: Analysing Twitter to Identify Barriers to Vaccination in a Low Uptake Region of the UK. Front Digit Health 2021;3:804855 [doi: 10.3389/fdgth.2021.804855] [Medline: 35141699]

114. Kalichman S, Eaton L, Earnshaw V, Brousseau N. Faster than warp speed: early attention to COVD-19 by anti-vaccine groups on Facebook. J Public Health (Oxf) 2022 Mar 07;44:e96-e105 [doi: 10.1093/pubmed/fdab093] [Medline: 33837428]

115. Wawrzuta D, Klejdysz J, Jaworski M, Gotlib J, Panczyk M. Attitudes toward COVID-19 Vaccination on Social Media: A Cross-Platform Analysis. Vaccines (Basel) 2022 Jul 27;10:1190 [doi: 10.3390/vaccines10081190] [Medline: 35893839]

117. Roozenbeek J, Schneider CR, Dryhurst S, Kerr J, Freeman ALJ, Recchia G, et al. Susceptibility to misinformation about COVID-19 around the world. R Soc Open Sci 2020 Oct;7:201199 [doi: 10.1098/rsos.201199] [Medline: 33204475]

120. Basch CH, Hillyer GC, Zagnit EA, Basch CE. YouTube coverage of COVID-19 vaccine development: implications for awareness and uptake. Hum Vaccin Immunother 2020 Nov 01;16:2582-2585 [doi: 10.1080/21645515.2020.1790280] [Medline: 32701403]

121. Chan C, Sounderajah V, Daniels E, Acharya A, Clarke J, Yalamanchili S, et al. The Reliability and Quality of YouTube Videos as a Source of Public Health Information Regarding COVID-19 Vaccination: Cross-sectional Study. JMIR Public Health Surveill 2021 Jul 08;7:e29942 [doi: 10.2196/29942] [Medline: 34081599]

122. Criss S, Nguyen TT, Norton S, Virani I, Titherington E, Tillmanns EL, et al. Advocacy, Hesitancy, and Equity: Exploring U.S. Race-Related Discussions of the COVID-19 Vaccine on Twitter. Int J Environ Res Public Health 2021 May 26;18:5693 [doi: 10.3390/ijerph18115693] [Medline: 34073291]
