# Supplementary material for: The Prevalence, Features, Influencing Factors, and Solutions for COVID-19 Vaccine Misinformation: Systematic Review
Source: JMIR Public Health Surveill. 2023 Jan 11;9:e40201. doi: 10.2196/40201 (PMC9838721; doi:10.2196/40201)
Supplement: Multimedia Appendix 6 [file publichealth_v9i1e40201_app6.docx]

##

## Appendix6. Proposed solutions to address misinformation in each study

| Reference | Category | Details |
| --- | --- | --- |
| Eshel et al., 2022 [89] | Disseminating trustworthy information | enhance vaccinations should be made only by experts or public opinion leaders who are trusted by specific communities |
| Andrade et al.,2021 [93] | Supporting the target audiences of misinformation | devise programs that provide special vaccination incentives to members of marginalized ethnic communities. |
| Garcia et al., 2021 [39] | Supporting the target audiences of misinformation | Participatory approaches that engage Latino families, lay health workers, and advocates can help address these gaps in health literacy |
| Gesualdo et al.,2022 [40] | Supporting the target audiences of misinformation | tailored, data-based communication strategies |
| An et al., 2021 [83] | Supporting the target audiences of misinformation | Encouraging more active information-seeking |
| Bíró‑Nagy and Szász, 2022 [32] | Supporting the target audiences of misinformation | these messages should be primarily targeted to young people, |
| Caycho-Rodríguez et al.,2022 [98] | Identifying misinformation; Disseminating trustworthy information | emphasize to track and identify misinformation about vaccines as a way to address these concerns with evidence-based information and ‘immunize’ people against misinformation |
| Jemielniak and Krempovych, 2021 [79] | Regulating producers and distributors | continuous monitoring of coordinated networks is required to detect and reduce misinformation in the public health discourse |
| Kumar et al., 2022 [109] | Disseminating trustworthy information | Training of media professionals and strategies for responsible reporting |
| Lin et al., 2022 [100] | Identifying misinformation; Supporting the target audiences of misinformation | 1) Fact-checking and improving individual information literacy, effective information and health intervention policies shall also address the impacts.  2) cooperation between scientific experts and the tech giants managing social media platforms may help to battle misinformation |
| Kricorian et al., 2022 [31] | Supporting the target audiences of misinformation; Disseminating trustworthy information | Portraying accurate COVID-19 information, especially regarding vaccines, in ways understandable to individuals from a variety of socioeconomic and educational backgrounds |
| Lurie et al., 2022 [54] | Disseminating trustworthy information | 1) better collaboration between media outlets and the public health/scientific communities might help ensure that journalists reporting on vaccines understand—and can accurately communicate 2) media outlets could work with public health organisations to generate an inclusive list of vetted science communicators and genuine public health experts who could serve as sources and/or review vaccine-relatedcontent to ensure that it is scientifically and medically accurate 3) conduct trainings for science and medical reporters |
| Kant, et al., 2021 [45] | Disseminating trustworthy information | A communication helpline should be developed to explain their fears and doubts about vaccines and gain insights into the situation. |
| Mahmud et al., 2021 [95] | Disseminating trustworthy information | 1) removing false social media contents, supplying impartial, accurate and realistic information about the COVID vaccine 2) traditional information channels such as the local public library and the district information office for disseminating vaccine-related information |
| Lamptey et al., 2022 [50] | Supporting the target audiences of misinformation | Health education interventions should target sociodemographic groups or areas in Africa that are identified as a priority |
| Li et al., 2022 [52] | Cutting production and distribution | Government to: 1) implementing mandates surrounding medical misinformation on social media platforms; 2) leverage these platforms to disseminate high-quality content to combat misinformation |
| Okoro et al., 2021 [58] | Supporting the target audiences of misinformation; Disseminating trustworthy information | 1) Endorsement of vaccine efcacy and safety by scientists, clinicians, community leaders, and peers 2) Targeted eforts at community education (open forums, one-on-one outreach, multiple media) |
| Ngai et al., 2022 [72] | Identifying misinformation; Cutting production and distribution | 1) Systematic monitoring of the antivaccine misinformation circulating on social media has to be undertaken. 2) To discern real news and misinformation, social media platforms or fact-checkers should focus not only on the content but also how it is conveyed by paying more attention to the writing strategies used in such posts. |
| Obreja et al., 2022 [57] | Disseminating trustworthy information | possible interventions by scientists, health experts and communication experts must be performed simultaneously |
| Neely et al., 2021 [56] | Disseminating trustworthy information | Public health professionals will need to become increasingly savvy in their use of social media to anticipate, identify, and respond to health-related misinformation |
| Wawrzuta et al., 2022 [115] | Disseminating trustworthy information | educational campaigns should consider these distinctions, focusing more on the platforms popular among adolescents |
| Wonodi et al., 2022 [66] | Disseminating trustworthy information | Targeted awareness campaigns and messages that deliver transparent information about COVID-19 vaccine |
| Wong et al., 2021 [28] | Disseminating trustworthy information | Findings of the COVID-19 vaccine clinical trials should be published in order to cease the widespread concerns |
| Ginossar et al., 2022 [123] | Identifying misinformation | 1) paramount that more attention be given to censoring anti-vaccination conten 2) interventions should not focus on one social media platform; rather, they should consider and integrate cross-platform |
| Wang et al., 2022 [63] | Disseminating trustworthy information | 1) educate people that vaccine induced immunity is safer than natural immunity against COVID-19; |
| Watermeyer et al., 2022 [64] | Supporting the target audiences of misinformation | Providing reliable and easy-to-understand messaging, engaging with the public and assisting ordinary people to navigate the enormous amount of reliable, and not so reliable, information |
| Jiang et al.,2021 [81] | Disseminating trustworthy information | 1) First, public health professionals should timely and appropriately address the public needs for vaccine-related information. 2) health communication may differentiate communication strategies for episodic and periodic themes. 3) Health organizations and health professionals should make more systematic and organized efforts to address antivaccination content and other vaccine-related misinformation. |
| Hammad et al., 2022 [88] | Disseminating trustworthy information | targeted campaigns to combat misconceptions, and given the rates of vaccine hesitancy |
| Criss et al., 2021 [122] | Disseminating trustworthy information | Partner with leader on social media and give exemplary messaging expressing vaccine support |
| Herrera-Peco et al., 2021 [15] | Identifying disinformation | Develop public health surveillance program led by health care organization allowing a rapid response |
| Griffith et al., 2021 [41] | Supporting the target audiences of disinformation;  Disseminating trustworthy information | 1. Give targeting vaccine literacy: how vaccines work, why they are safe, and no steps were missed in development. 2. Have trusted physicians to emphasize that vaccines are rooted in science and not politics. |
| Islam et al., 2021 [85] | Identifying disinformation;  Disseminating trustworthy information | 1. Public health agencies to monitor and track the most frequently shared misinformation 2. Use websites providing evidence-based information; Following the bottom-up approach; risk communication about disease risks, the role of vaccine and known side effects |
| Hughes et al., 2021 [73] | Disseminating trustworthy information | Coding studies and counter-message testing be undertaken prior to the launch of public health campaigns addressing anti-vaccine |
| Larrondo-Ureta et al., 2021 [113] | Disseminating trustworthy information | each user in the task of creating and disseminating messages continues to be the best antidote to misinformation in times of infodemic |
| Hernández-García et al., 2021 [43] | Supporting the target audiences of disinformation;  Disseminating trustworthy information | 1. Debunk false claims 2. Information be should be given through diverse mediums, mainly by health professionals |
| Jennings et al., 2021 [108] | Cutting production and distribution;  Supporting the target audiences of disinformation | 1. An engaging web led by government; advertisers boycott harmful content; companies company sensor content; 2. Sites use flags to help identify |
| Ebrahimi et al., 2021 [38] | Identifying disinformation;  Disseminating trustworthy information | 1. Identify outlets and channels of misinformation by health officer  2. Effective communication to address emotional responses and cognitive biases by appealing to altruism, framing vaccination as a step to society as a whole, creating a sense of communion in the battle |
| Chan et al., 2021 [121] | Cutting production and distribution;  Disseminating trustworthy information | 1. Social media to remove the misinformation rapidly 2. Disseminate authoritative messages regarding vaccine safety; mobilize medical professionals; the importance of brand recognition or marketing in attracting audiences |
| Jensen et al., 2021 [97] | Regulating producers and distributors;  Disseminating trustworthy information | Policies and (science) communication on the governmental and institutional level to establish and maintain long-term, mutually beneficial relationships of trust with diverse publics |
| Aloweidi et al., 2021 [102] | Regulating producers and distributors;  Supporting the target audiences of disinformation;  Disseminating trustworthy information | 1. Legal action taken by the government and the public security directorate 2. Social media awareness campaigns; adequate funding for national medical studies to prove the safety and efficacy 3. Short scientific videos to encourage vaccination |
| Arshad et al., 2021 [74] | Supporting the target audiences of disinformation | Implementing national public awareness campaigns at the government level |
| Baines et al., 2021 [110] | Cutting production and distribution;  Supporting the target audiences of disinformation | 1. Platform to use moderated content policy 2. Vaccine hesitant individuals need to be sensitized and included in civil dialogues online and offline |
| Basch et al., 2020 [120] | Disseminating trustworthy information | Public health officials to learn how to produce accurate videos that are appealing |
| Sallam et al., 2021 [60] | Disseminating trustworthy information | Delivering clear, timely, and evidence-based messages through legitimate channels; collaboration of the scientific community/experts and media sources |
| Romer and Jamieson, 2021 [1] | Disseminating trustworthy information | Use popular source of information (use of mainstream television/Use of broadcast television) |
| Roozenbeek et al., 2020 [117] | Disseminating trustworthy information | Post hoc corrections may backfire, pre-emptive refutations of conspiracy theories through a process known as ‘cognitive inoculation’ can be effective at reducing belief in misinformation. |
| Wawrzuta et al., 2021 [65] | Disseminating trustworthy information | Public health authorities to publicly emphasize that vaccines registered have undergone the entire registration process. |
| Dereje et al., 2021 [37] | Supporting the target audiences of disinformation | Providing the community with health education and consistent government efforts |
| Kumar et al., 2022 [48] | Cutting production and distribution;  Supporting the target audiences of disinformation;  Disseminating trustworthy information | 1. More effective moderation policies in company; 2. Conduct tailored interventions: skeptics or critics with more evidence-based information; communications campaigns nudging individuals toward accurate vaccine information 3. Scientists be more communicative |
| Manby et al., 2021 [94] | Supporting the target audiences of disinformation | Directly engage with vaccine-hesitant sub-groups, understanding how a history of exclusion and racism |
| Sharevski et al., 2021 [104] | Cutting production and distribution | Interstitial warnings or cover warnings in our study in that they required individuals to click through to continue |
| Dai et al.,2022 [128] | Supporting the target audiences of misinformation | Communication practitioners and campaign designers could consider providing incentives to the audience to share the message and openly express their endorsement. |
| Lu and Zhong, 2022 [125] | Cutting production and distribution | Partisan media should consider how the evidence can be strategically presented, so as to tailor to different ideological groups. |
| Helfers et al., 2022 [118] | Cutting production and distribution | The social listening tool of the WHO could be useful to adapt communication strategies to the current concerns about the vaccine circulating among the public. |
| Talabi et al., 2022 [126] | Cutting production and distribution | Social media-based counselling can be an effective tool for health communication and health promotion during pandemics like COVID-19. |
| Thaker and Subramanian, 2021 [75] | Disseminating trustworthy information | 1)Misinformation correction campaigns should not only focus on outright misinformation but also on vaccine hesitancy 2) Public health officials should not hesitate to transparently inform people of the limitations of vaccinations either, providing them with an authoritative resource rather than driving them to alternative |
| Vivion et al., 2022 [119] | Disseminating trustworthy information | Public health authorities to integrate infodemiology and infoveillance expertise to target the more impactful misinformation messages. |

## References:

1. Romer D, Jamieson KH. Patterns of Media Use, Strength of Belief in COVID-19 Conspiracy Theories, and the Prevention of COVID-19 From March to July 2020 in the United States: Survey Study. J Med Internet Res 2021 Apr 27;23:e25215 [doi: 10.2196/25215] [Medline: 33857008]

15. Herrera-Peco I, Jiménez-Gómez B, Romero Magdalena CS, Deudero JJ, García-Puente M, Benítez De Gracia E, et al. Antivaccine Movement and COVID-19 Negationism: A Content Analysis of Spanish-Written Messages on Twitter. Vaccines (Basel) 2021 Jun 15;9:656 [doi: 10.3390/vaccines9060656] [Medline: 34203946]

28. Wong LP, Lin Y, Alias H, Bakar SA, Zhao Q, Hu Z. COVID-19 Anti-Vaccine Sentiments: Analyses of Comments from Social Media. Healthcare (Basel) 2021 Dec 09;9:1530 [doi: 10.3390/healthcare9111530] [Medline: 34828576]

31. Kricorian K, Civen R, Equils O. COVID-19 vaccine hesitancy: misinformation and perceptions of vaccine safety. Hum Vaccin Immunother 2022 Dec 31;18:1950504 [doi: 10.1080/21645515.2021.1950504] [Medline: 34325612]

32. Bíró-Nagy A, Szászi áJ. The roots of COVID-19 vaccine hesitancy: evidence from Hungary. J Behav Med 2022 May 14:1-16 [doi: 10.1007/s10865-022-00314-5] [Medline: 35567729]

37. Dereje N, Tesfaye A, Tamene B, Alemeshet D, Abe H, Tesfa N, et al. COVID-19 vaccine hesitancy in Addis Ababa, Ethiopia: a mixed-method study. BMJ Open 2022 May 30;12:e052432 [doi: 10.1136/bmjopen-2021-052432] [Medline: 35636790]

38. Ebrahimi OV, Johnson MS, Ebling S, Amundsen OM, Halsøy Ø, Hoffart A, et al. Risk, Trust, and Flawed Assumptions: Vaccine Hesitancy During the COVID-19 Pandemic. Front Public Health 2021;9:700213 [doi: 10.3389/fpubh.2021.700213] [Medline: 34277557]

39. Garcia J, Vargas N, de la Torre C, Magana Alvarez M, Clark JL. Engaging Latino Families About COVID-19 Vaccines: A Qualitative Study Conducted in Oregon, USA. Health Educ Behav 2021 Dec;48:747-757 [doi: 10.1177/10901981211045937] [Medline: 34596462]

40. Gesualdo F, Parisi L, Croci I, Comunello F, Parente A, Russo L, et al. How the Italian Twitter Conversation on Vaccines Changed During the First Phase of the Pandemic: A Mixed-Method Analysis. Front Public Health 2022;10:824465 [doi: 10.3389/fpubh.2022.824465] [Medline: 35664110]

41. Griffith J, Marani H, Monkman H. COVID-19 Vaccine Hesitancy in Canada: Content Analysis of Tweets Using the Theoretical Domains Framework. J Med Internet Res 2021 Apr 13;23:e26874 [doi: 10.2196/26874] [Medline: 33769946]

43. Hernández-García I, Gascón-Giménez I, Gascón-Giménez A, Giménez-Júlvez T. Information in Spanish on YouTube about Covid-19 vaccines. Hum Vaccin Immunother 2021 Nov 02;17:3916-3921 [doi: 10.1080/21645515.2021.1957416] [Medline: 34375570]

45. Kant R, Varea RR, Titifanue J. COVID-19 vaccine online misinformation in Fiji: Preliminary findings. Pacific Journalism Review 2022;27:47-62 [doi: 10.24135/pjr.v27i1&2.1189]

48. Kumar N, Corpus I, Hans M, Harle N, Yang N, McDonald C, et al. COVID-19 vaccine perceptions in the initial phases of US vaccine roll-out: an observational study on reddit. BMC Public Health 2022 Mar 07;22:446 [doi: 10.1186/s12889-022-12824-7] [Medline: 35255881]

50. Lamptey E, Senkyire EK, Dorcas S, Benita DA, Boakye EO, Ikome T, et al. Exploring the myths surrounding the COVID-19 vaccines in Africa: the study to investigate their impacts on acceptance using online survey and social media. Clin Exp Vaccine Res 2022 May;11:193-208 [doi: 10.7774/cevr.2022.11.2.193] [Medline: 35799880]

52. Li HOY, Pastukhova E, Brandts-Longtin O, Tan MG, Kirchhof MG. YouTube as a source of misinformation on COVID-19 vaccination: a systematic analysis. BMJ Glob Health 2022 Mar;7:e008334 [doi: 10.1136/bmjgh-2021-008334] [Medline: 35264318]

54. Lurie P, Adams J, Lynas M, Stockert K, Carlyle RC, Pisani A, et al. COVID-19 vaccine misinformation in English-language news media: retrospective cohort study. BMJ Open 2022 Jun 01;12:e058956 [doi: 10.1136/bmjopen-2021-058956] [Medline: 35649595]

56. Neely SR, Eldredge C, Ersing R, Remington C. Vaccine Hesitancy and Exposure to Misinformation: a Survey Analysis. J Gen Intern Med 2022 Jan;37:179-187 [doi: 10.1007/s11606-021-07171-z] [Medline: 34671900]

57. Obreja DM. Narrative communication regarding the Covid-19 vaccine: a thematic analysis of comments on Romanian official Facebook page "RO Vaccinare". SN Soc Sci 2022;2:119 [doi: 10.1007/s43545-022-00427-3] [Medline: 35875608]

58. Okoro O, Kennedy J, Simmons G, Vosen EC, Allen K, Singer D, et al. Exploring the Scope and Dimensions of Vaccine Hesitancy and Resistance to Enhance COVID-19 Vaccination in Black Communities. J Racial Ethn Health Disparities 2022 Dec;9:2117-2130 [doi: 10.1007/s40615-021-01150-0] [Medline: 34553340]

60. Sallam M, Dababseh D, Eid H, Hasan H, Taim D, Al-Mahzoum K, et al. Low COVID-19 Vaccine Acceptance Is Correlated with Conspiracy Beliefs among University Students in Jordan. Int J Environ Res Public Health 2021 Mar 01;18:2407 [doi: 10.3390/ijerph18052407] [Medline: 33804558]

63. Wang CW, de Jong EP, Faure JA, Ellington JL, Chen CHS, Chan CC. A matter of trust: a qualitative comparison of the determinants of COVID-19 vaccine hesitancy in Taiwan, the United States, the Netherlands, and Haiti. Hum Vaccin Immunother 2022 Nov 30;18:2050121 [doi: 10.1080/21645515.2022.2050121] [Medline: 35349382]

64. Watermeyer J, Scott M, Kapueja L, Ware LJ. To trust or not to trust: an exploratory qualitative study of personal and community perceptions of vaccines amongst a group of young community healthcare workers in Soweto, South Africa. Health Policy Plan 2022 Oct 12;37:1167-1176 [doi: 10.1093/heapol/czac060] [Medline: 35880606]

65. Wawrzuta D, Jaworski M, Gotlib J, Panczyk M. What Arguments against COVID-19 Vaccines Run on Facebook in Poland: Content Analysis of Comments. Vaccines (Basel) 2021 May 10;9:481 [doi: 10.3390/vaccines9050481] [Medline: 34068500]

66. Wonodi C, Obi-Jeff C, Adewumi F, Keluo-Udeke SC, Gur-Arie R, Krubiner C, et al. Conspiracy theories and misinformation about COVID-19 in Nigeria: Implications for vaccine demand generation communications. Vaccine 2022 Mar 18;40:2114-2121 [doi: 10.1016/j.vaccine.2022.02.005] [Medline: 35153088]

72. Ngai CSB, Singh RG, Yao L. Impact of COVID-19 Vaccine Misinformation on Social Media Virality: Content Analysis of Message Themes and Writing Strategies. J Med Internet Res 2022 Jul 06;24:e37806. [doi: 10.2196/37806] [Medline: 35731969]

73. Hughes B, Miller-Idriss C, Piltch-Loeb R, Goldberg B, White K, Criezis M, et al. Development of a Codebook of Online Anti-Vaccination Rhetoric to Manage COVID-19 Vaccine Misinformation. Int J Environ Res Public Health 2021 Jul 15;18:7556 [doi: 10.3390/ijerph18147556] [Medline: 34300005]

74. Arshad MS, Hussain I, Mahmood T, Hayat K, Majeed A, Imran I, et al. A National Survey to Assess the COVID-19 Vaccine-Related Conspiracy Beliefs, Acceptability, Preference, and Willingness to Pay among the General Population of Pakistan. Vaccines (Basel) 2021 Jul 01;9:720 [doi: 10.3390/vaccines9070720] [Medline: 34358136]

75. Thaker J, Subramanian A. Exposure to COVID-19 Vaccine Hesitancy Is as Impactful as Vaccine Misinformation in Inducing a Decline in Vaccination Intentions in New Zealand: Results from Pre-Post Between-Groups Randomized Block Experiment. Front. Commun 2021 Aug 19;6:721982. [doi: 10.3389/fcomm.2021.721982]

79. Jemielniak D, Krempovych Y. An analysis of AstraZeneca COVID-19 vaccine misinformation and fear mongering on Twitter. Public Health 2021 Nov;200:4-6 [doi: 10.1016/j.puhe.2021.08.019] [Medline: 34628307]

81. Jiang LC, Chu TH, Sun M. Characterization of Vaccine Tweets During the Early Stage of the COVID-19 Outbreak in the United States: Topic Modeling Analysis. JMIR Infodemiology 2021;1:e25636 [doi: 10.2196/25636] [Medline: 34604707]

83. An L, Russell DM, Mihalcea R, Bacon E, Huffman S, Resnicow K. Online Search Behavior Related to COVID-19 Vaccines: Infodemiology Study. JMIR Infodemiology 2021;1:e32127 [doi: 10.2196/32127] [Medline: 34841200]

85. Islam MS, Kamal AM, Kabir A, Southern DL, Khan SH, Hasan SMM, et al. COVID-19 vaccine rumors and conspiracy theories: The need for cognitive inoculation against misinformation to improve vaccine adherence. PLoS One 2021;16:e0251605 [doi: 10.1371/journal.pone.0251605] [Medline: 33979412]

88. Hammad AM, Al-Qerem W, Abu Zaid A, Khdair SI, Hall FS. Misconceptions Related to COVID 19 Vaccines Among the Jordanian Population: Myth and Public Health. Disaster Med Public Health Prep 2022 Jun 08:1-8 [doi: 10.1017/dmp.2022.143] [Medline: 35673791]

89. Eshel Y, Kimhi S, Marciano H, Adini B. Conspiracy claims and secret intentions as predictors of psychological coping and vaccine uptake during the COVID-19 pandemic. J Psychiatr Res 2022 Jul;151:311-318 [doi: 10.1016/j.jpsychires.2022.04.042] [Medline: 35526447]

93. Andrade G. Covid-19 vaccine hesitancy, conspiracist beliefs, paranoid ideation and perceived ethnic discrimination in a sample of University students in Venezuela. Vaccine 2021 Nov 16;39:6837-6842 [doi: 10.1016/j.vaccine.2021.10.037] [Medline: 34711439]

94. Manby L, Dowrick A, Karia A, Maio L, Buck C, Singleton G, et al. Healthcare workers’ perceptions and attitudes towards the UK’s COVID-19 vaccination programme: a rapid qualitative appraisal. BMJ Open 2022 Feb 15;12:e051775. [doi: 10.1136/bmjopen-2021-051775]

95. Mahmud MR, Bin Reza R, Ahmed SZ. The effects of misinformation on COVID-19 vaccine hesitancy in Bangladesh. GKMC 2021 Oct 24:ahead-of-print. [doi: 10.1108/gkmc-05-2021-0080]

97. Jensen EA, Pfleger A, Herbig L, Wagoner B, Lorenz L, Watzlawik M. What Drives Belief in Vaccination Conspiracy Theories in Germany? Front. Commun 2021 May 25;6:105. [doi: 10.3389/fcomm.2021.678335]

98. Caycho-Rodríguez T, Ventura-León J, Valencia PD, Vilca LW, Carbajal-León C, Reyes-Bossio M, et al. What Is the Support for Conspiracy Beliefs About COVID-19 Vaccines in Latin America? A Prospective Exploratory Study in 13 Countries. Front. Psychol 2022 May 6;13:105. [doi: 10.3389/fpsyg.2022.855713]

100. Lin F, Chen X, Cheng EW. Contextualized impacts of an infodemic on vaccine hesitancy: The moderating role of socioeconomic and cultural factors. Information Processing & Management 2022 Sep;59:103013. [doi: 10.1016/j.ipm.2022.103013]

102. Aloweidi A, Bsisu I, Suleiman A, Abu-Halaweh S, Almustafa M, Aqel M, et al. Hesitancy towards COVID-19 Vaccines: An Analytical Cross-Sectional Study. Int J Environ Res Public Health 2021 May 12;18:5111 [doi: 10.3390/ijerph18105111] [Medline: 34065888]

104. Sharevski F, Alsaadi R, Jachim P, Pieroni E. Misinformation warnings: Twitter's soft moderation effects on COVID-19 vaccine belief echoes. Comput Secur 2022 Mar;114:102577 [doi: 10.1016/j.cose.2021.102577] [Medline:34934255]

108. Jennings W, Stoker G, Bunting H, Valgaresson VO, Gaskell J, Devine D, et al. Lack of Trust, Conspiracy Beliefs, and Social Media Use Predict COVID-19 Vaccine Hesitancy. Vaccines (Basel) 2021 Jun 03;9:593 [doi: 10.3390/vaccines9060593] [Medline: 34204971]

109. Kumar M, Madhumathi J, Gayathri K, A Rozario AG, Vijayaprabha R, Balusamy M, et al. Community voices around COVID-19 vaccine in Chennai, India: A qualitative exploration during early phase of vaccine rollout. Indian J Med Res 2022;155:451-460. [doi: 10.4103/ijmr.ijmr_668_22] [Medline: 35975352]

110. Baines A, Ittefaq M, Abwao M. #Scamdemic, #Plandemic, or #Scaredemic: What Parler Social Media Platform Tells Us about COVID-19 Vaccine. Vaccines (Basel) 2021 Apr 22;9:421 [doi: 10.3390/vaccines9050421] [Medline:33922343]

113. Larrondo-Ureta A, Fernández S, Morales-i-Gras J. Desinformación, vacunas y Covid-19. Análisis de la infodemia y la conversación digital en Twitter. RLCS 2021 Jun 07:1-18. [doi: 10.4185/rlcs-2021-1504]

115. Wawrzuta D, Klejdysz J, Jaworski M, Gotlib J, Panczyk M. Attitudes toward COVID-19 Vaccination on Social Media: A Cross-Platform Analysis. Vaccines (Basel) 2022 Jul 27;10:1190 [doi: 10.3390/vaccines10081190] [Medline: 35893839]

117. Roozenbeek J, Schneider CR, Dryhurst S, Kerr J, Freeman ALJ, Recchia G, et al. Susceptibility to misinformation about COVID-19 around the world. R Soc Open Sci 2020 Oct;7:201199 [doi: 10.1098/rsos.201199] [Medline: 33204475]

118. Helfers A, Ebersbach M. The differential effects of a governmental debunking campaign concerning COVID-19 vaccination misinformation. Journal of Communication in Healthcare 2022 Mar 17:1-9. [doi: 10.1080/17538068.2022.2047497]

119. Vivion M, Anassour Laouan Sidi E, Betsch C, Dionne M, Dubé E, Driedger SM, et al. Prebunking messaging to inoculate against COVID-19 vaccine misinformation: an effective strategy for public health. Journal of Communication in Healthcare 2022 Mar 04;15:232-242. [doi: 10.1080/17538068.2022.2044606]

120. Basch CH, Hillyer GC, Zagnit EA, Basch CE. YouTube coverage of COVID-19 vaccine development: implications for awareness and uptake. Hum Vaccin Immunother 2020 Nov 01;16:2582-2585 [doi: 10.1080/21645515.2020.1790280] [Medline: 32701403]

121. Chan C, Sounderajah V, Daniels E, Acharya A, Clarke J, Yalamanchili S, et al. The Reliability and Quality of YouTube Videos as a Source of Public Health Information Regarding COVID-19 Vaccination: Cross-sectional Study. JMIR Public Health Surveill 2021 Jul 08;7:e29942 [doi: 10.2196/29942] [Medline: 34081599]

122. Criss S, Nguyen TT, Norton S, Virani I, Titherington E, Tillmanns EL, et al. Advocacy, Hesitancy, and Equity: Exploring U.S. Race-Related Discussions of the COVID-19 Vaccine on Twitter. Int J Environ Res Public Health 2021 May 26;18:5693 [doi: 10.3390/ijerph18115693] [Medline: 34073291]

123. Ginossar T, Cruickshank IJ, Zheleva E, Sulskis J, Berger-Wolf T. Cross-platform spread: vaccine-related content, sources, and conspiracy theories in YouTube videos shared in early Twitter COVID-19 conversations. Hum Vaccin Immunother 2022 Dec 31;18:1-13 [doi: 10.1080/21645515.2021.2003647] [Medline: 35061560]

125. Lu S, Zhong L. From Believing to Sharingxamining the Effects of Partisan Media's Correction of COVID-19 Vaccine Misinformation. INTERNATIONAL JOURNAL OF COMMUNICATION. 2022 2022 2022 Jun 15:16.

126. Talabi F, Ugbor I, Talabi M, Ugwuoke J, Oloyede D, Aiyesimoju A, et al. Effect of a social media-based counselling intervention in countering fake news on COVID-19 vaccine in Nigeria. Health Promot Int 2022 Apr 29;37:daab140. [doi: 10.1093/heapro/daab140] [Medline: 34510187]

128. Dai Y, Jia W, Fu L, Sun M, Jiang LC. The effects of self-generated and other-generated eWOM in inoculating against misinformation. Telematics and Informatics 2022 Jul;71:101835. [doi: 10.1016/j.tele.2022.101835]
